# Supplementary figures and images for: Comprehensive analysis of Verticillium nonalfalfae in silico secretome uncovers putative effector proteins expressed during hop invasion
Source: PLoS One. 2018 Jun 12;13(6):e0198971. doi: 10.1371/journal.pone.0198971 (PMC5997321; doi:10.1371/journal.pone.0198971)

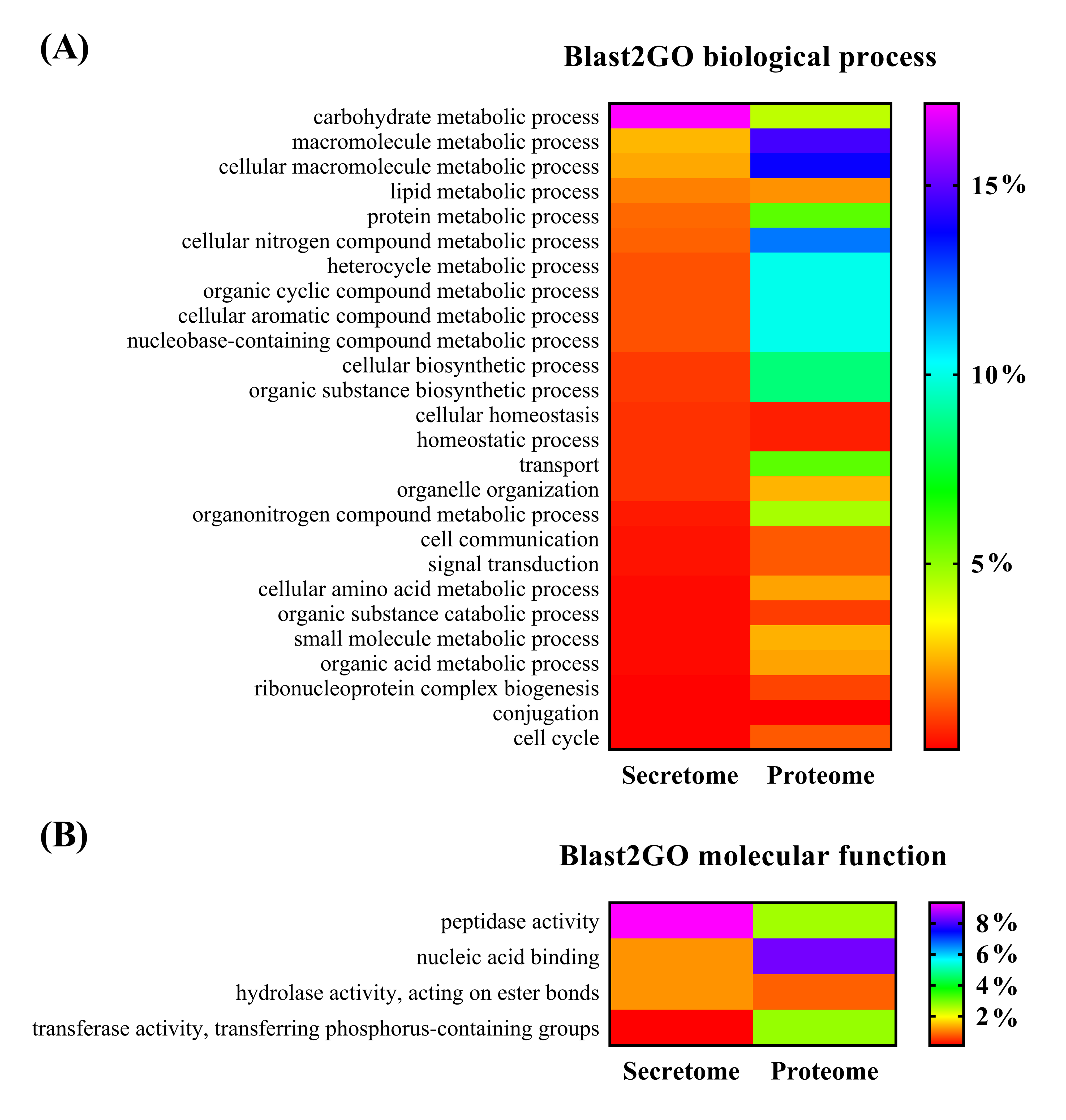

Supplement: S1 Fig — GO terms for biological process (A) and molecular function (B) are presented at GO level 4. Relative abundance of gene ontology (GO) terms determined by Blast2GO is expressed in percentages of predicted fungal secretome and proteome, respectively. (TIF) [file pone.0198971.s009.tif]

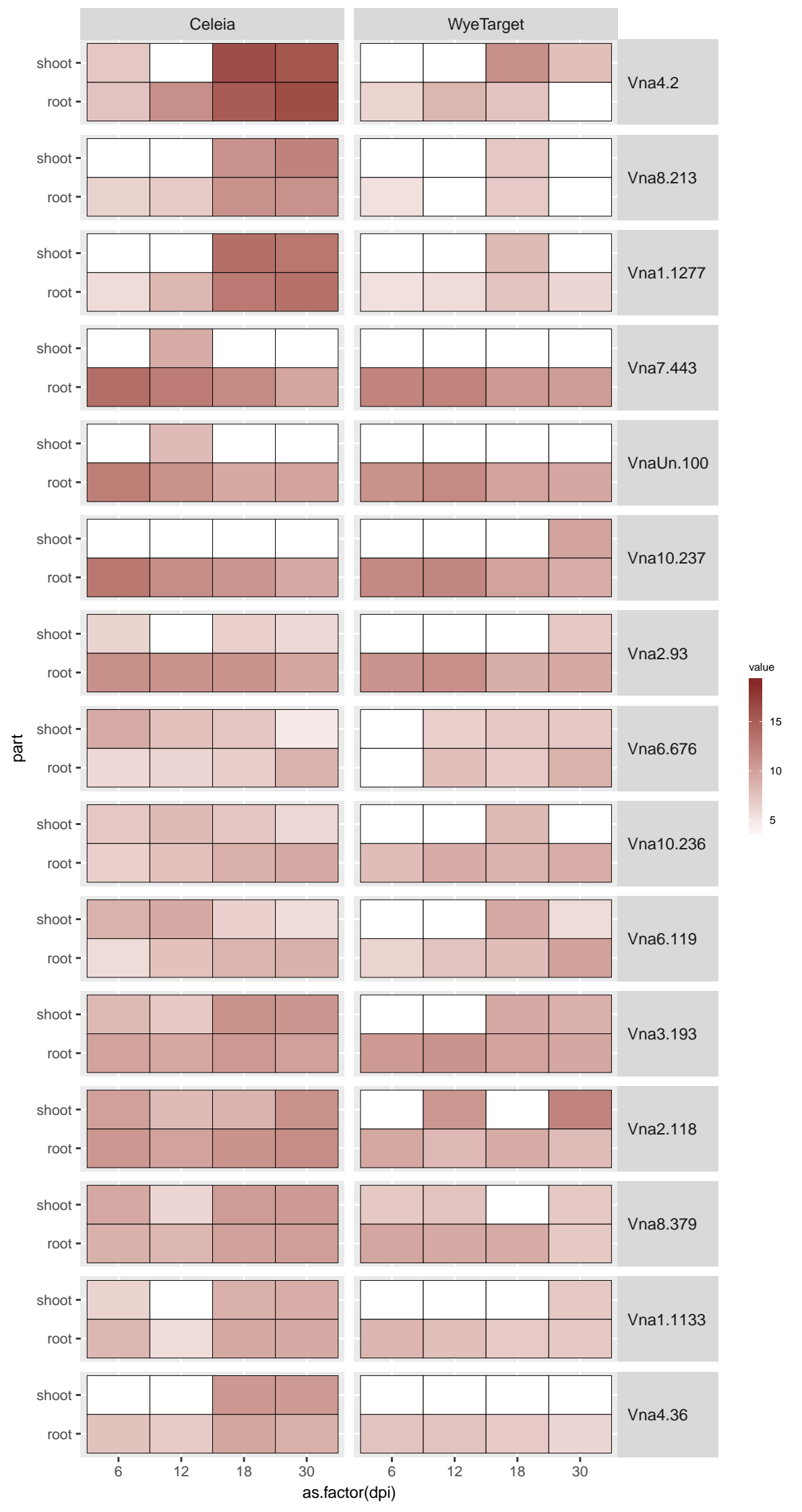

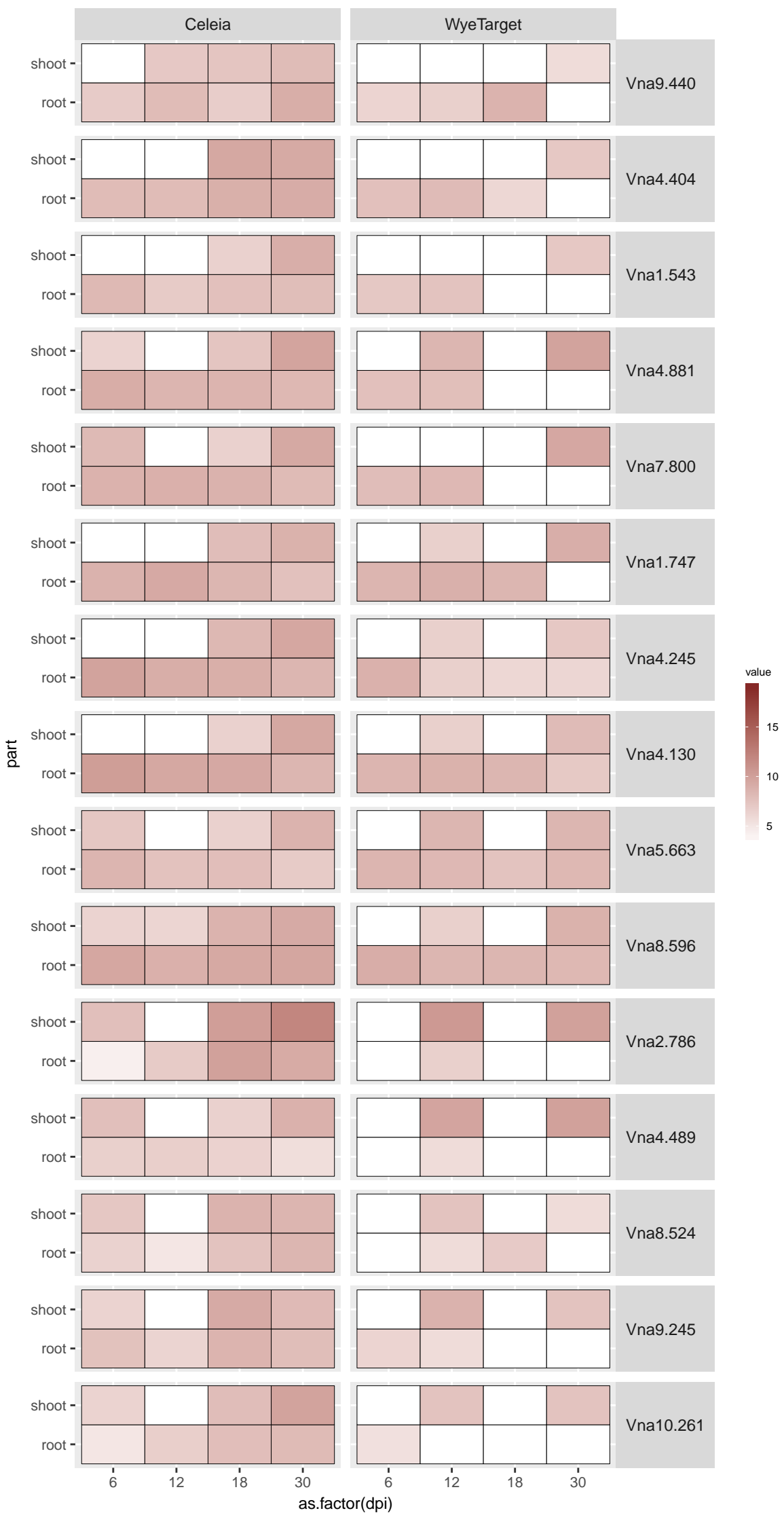

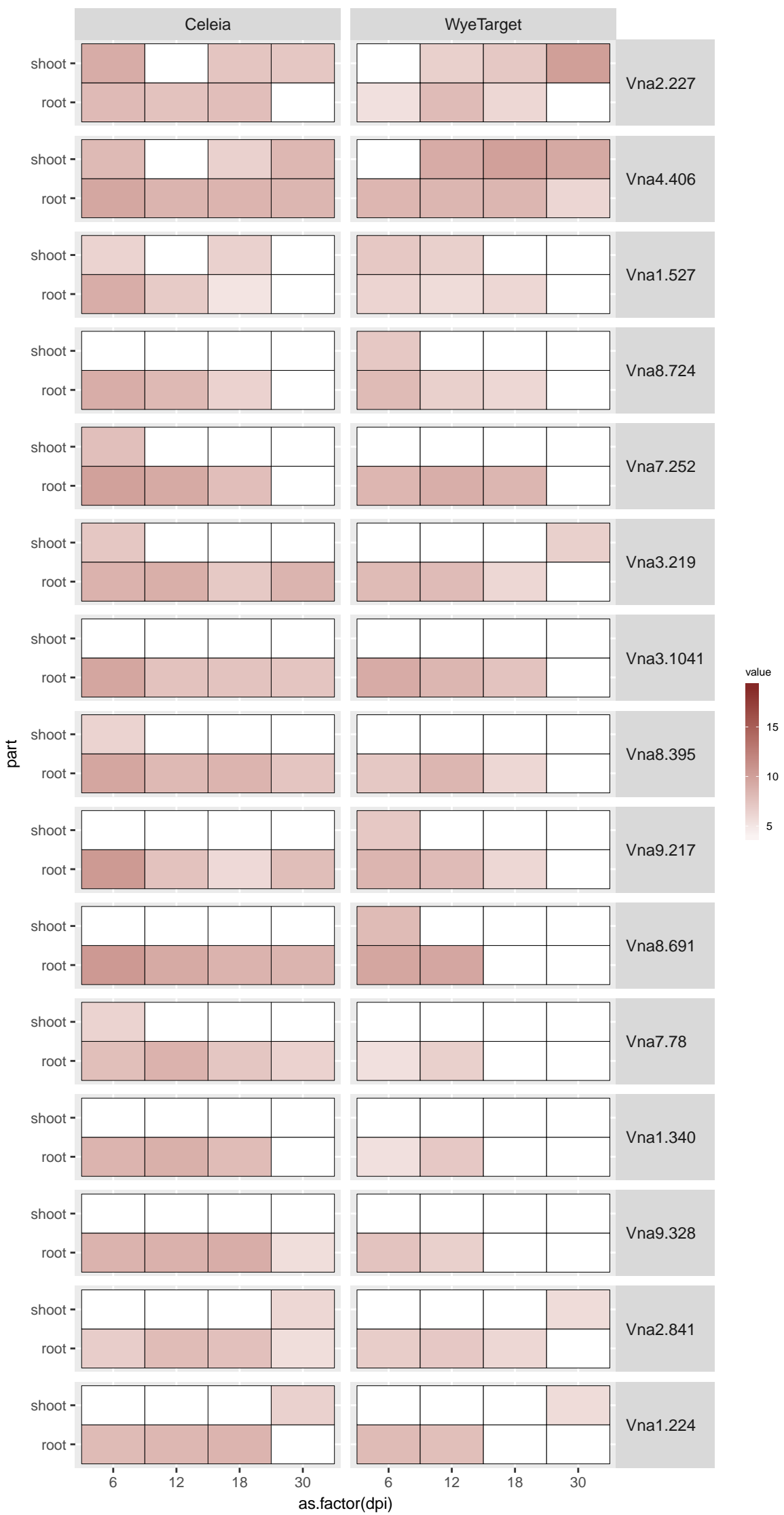

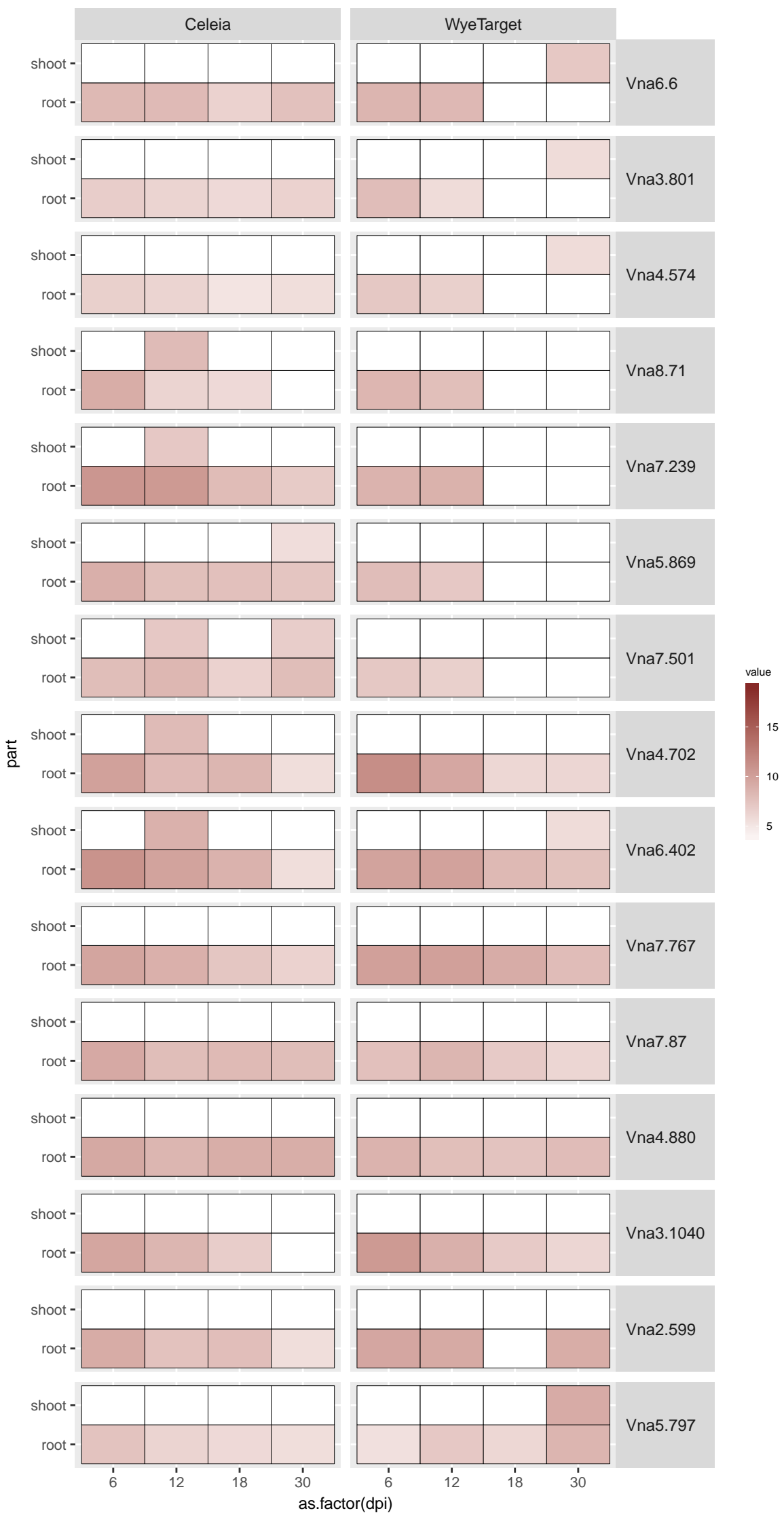

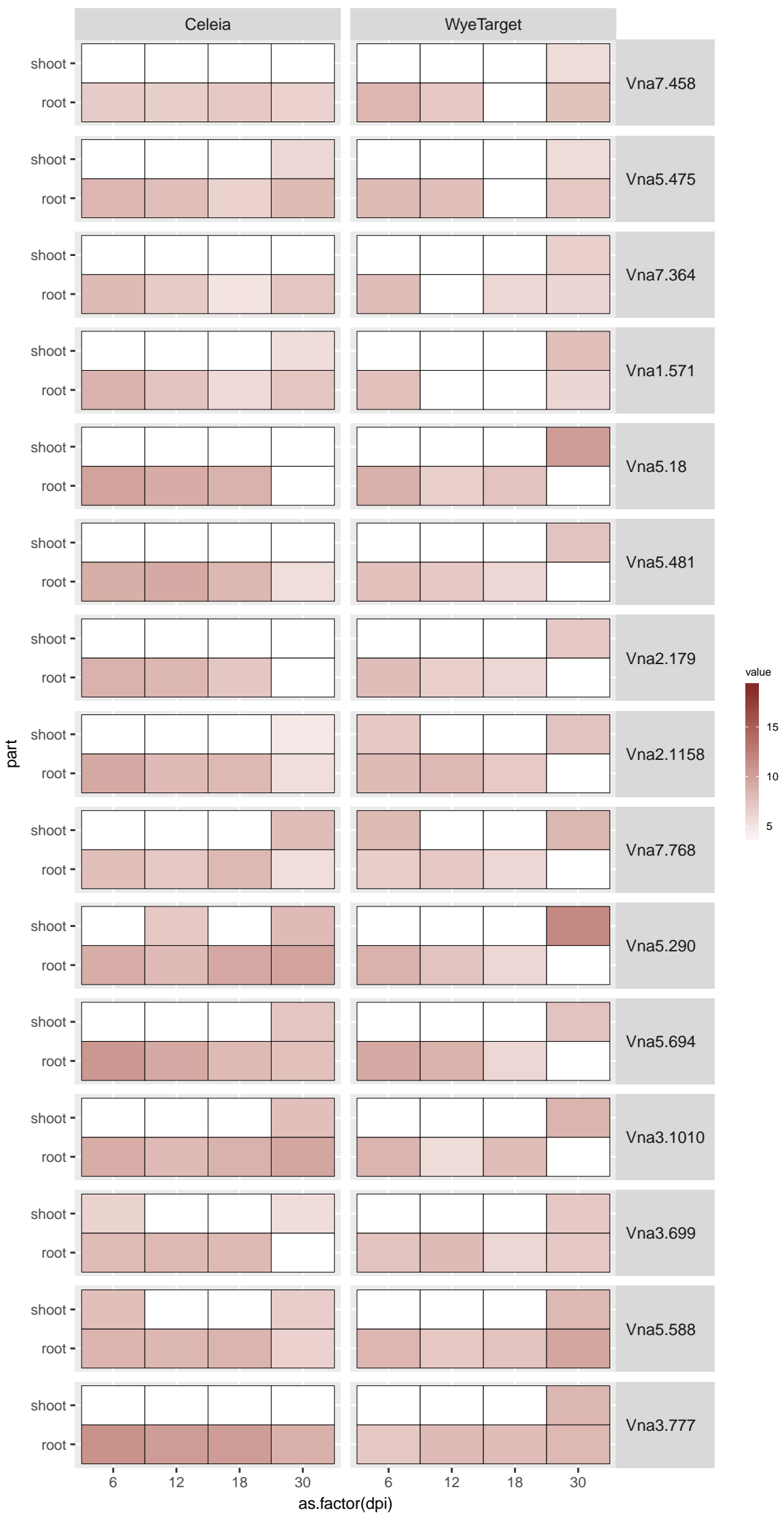

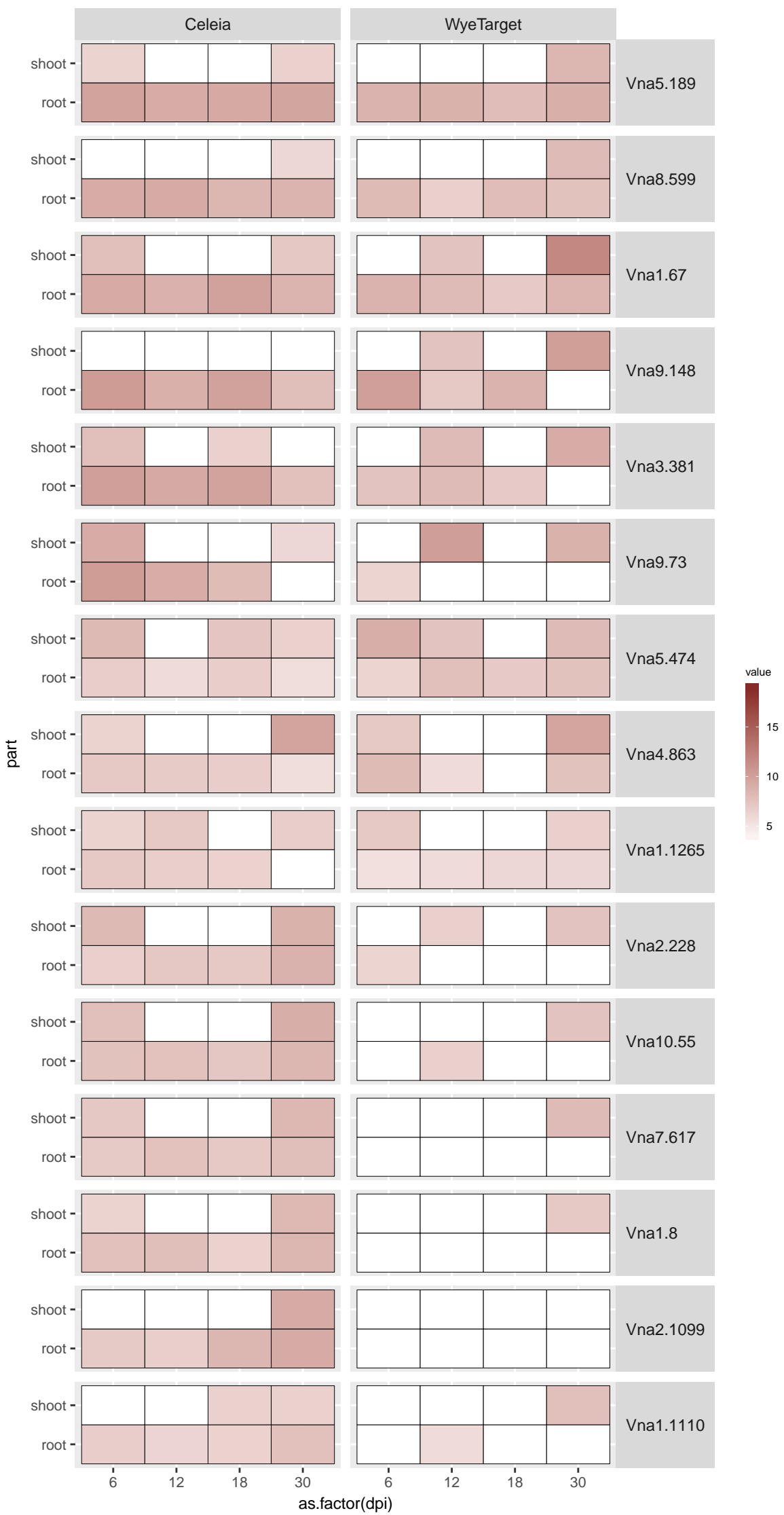

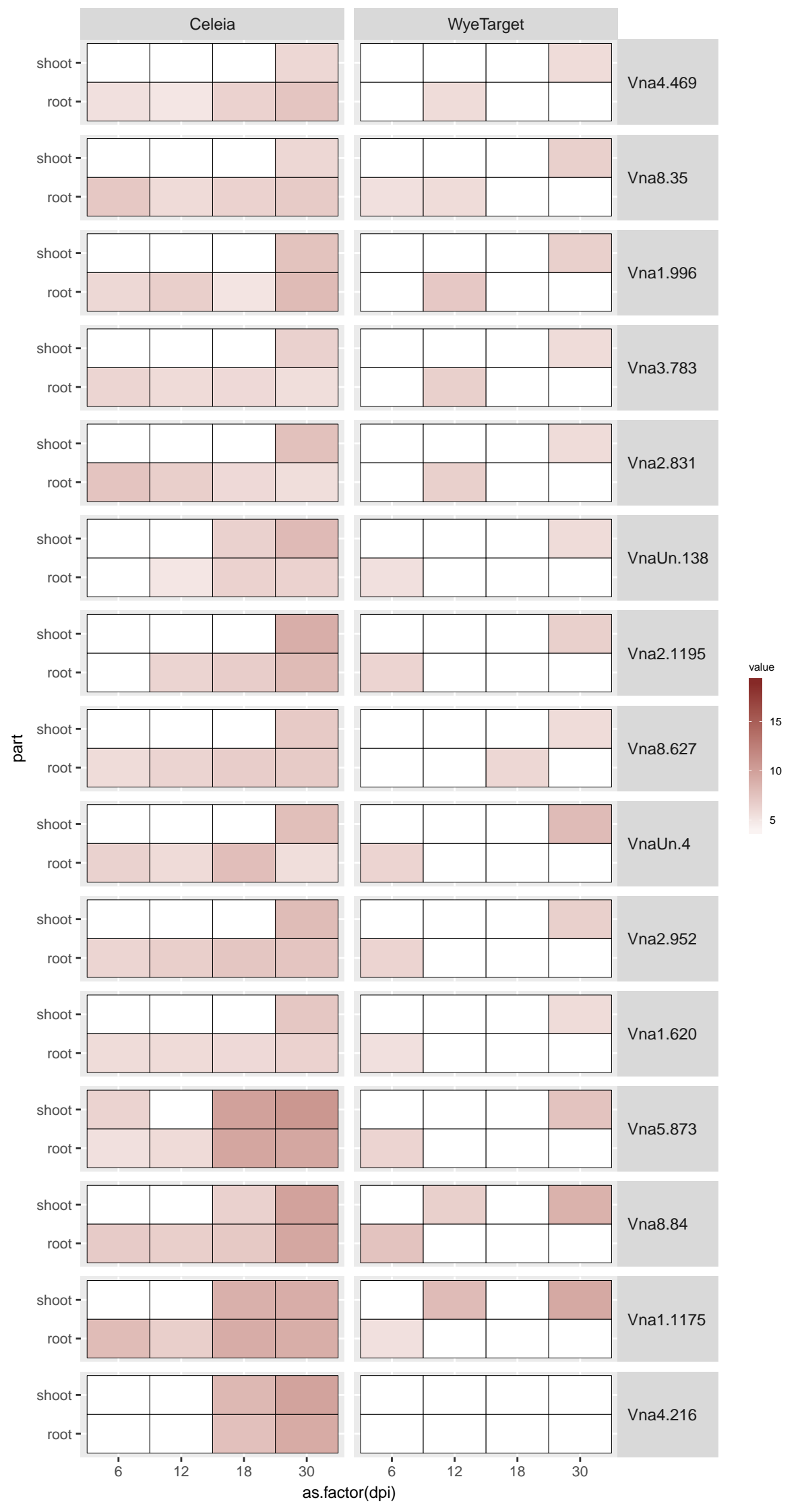

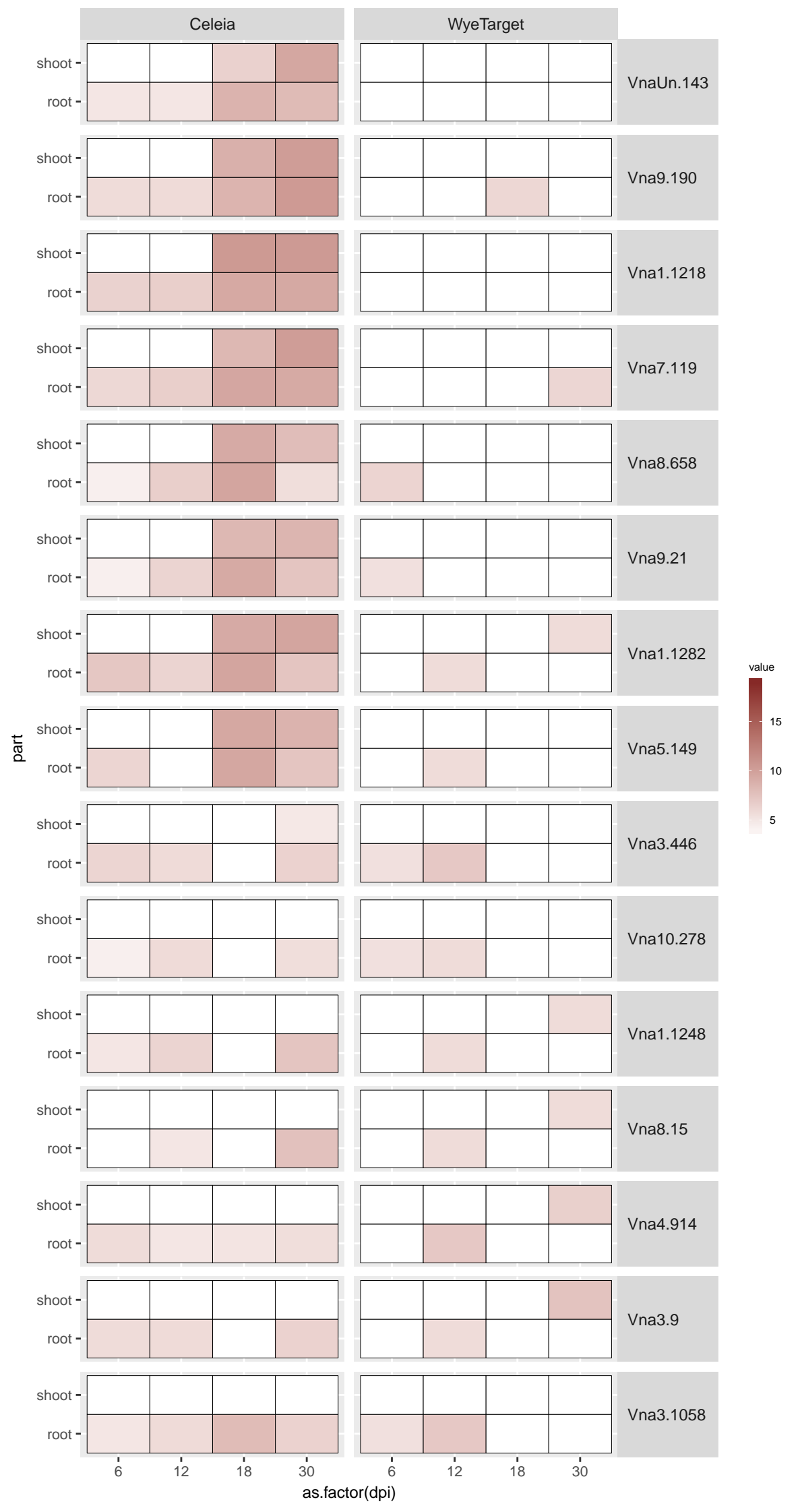

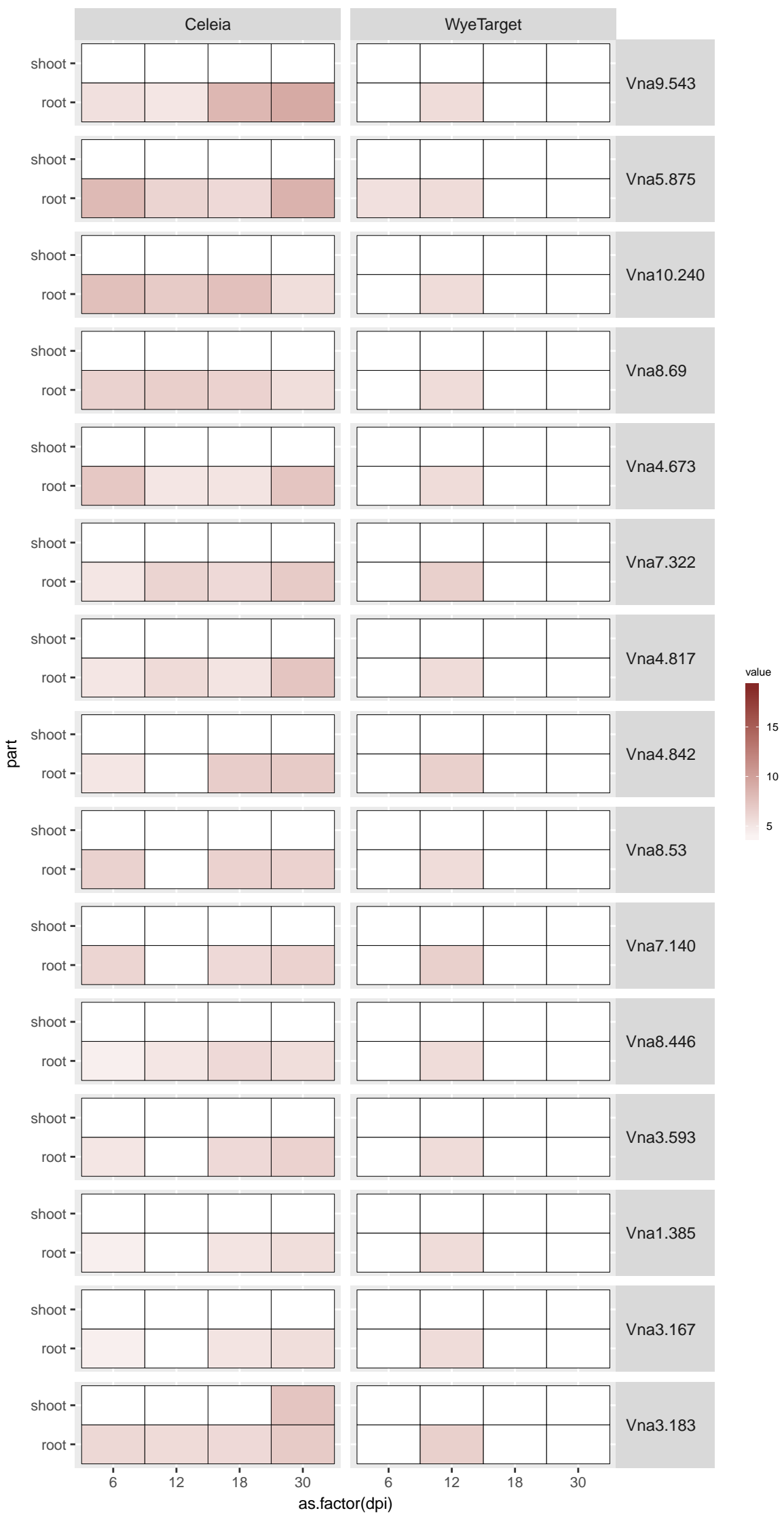

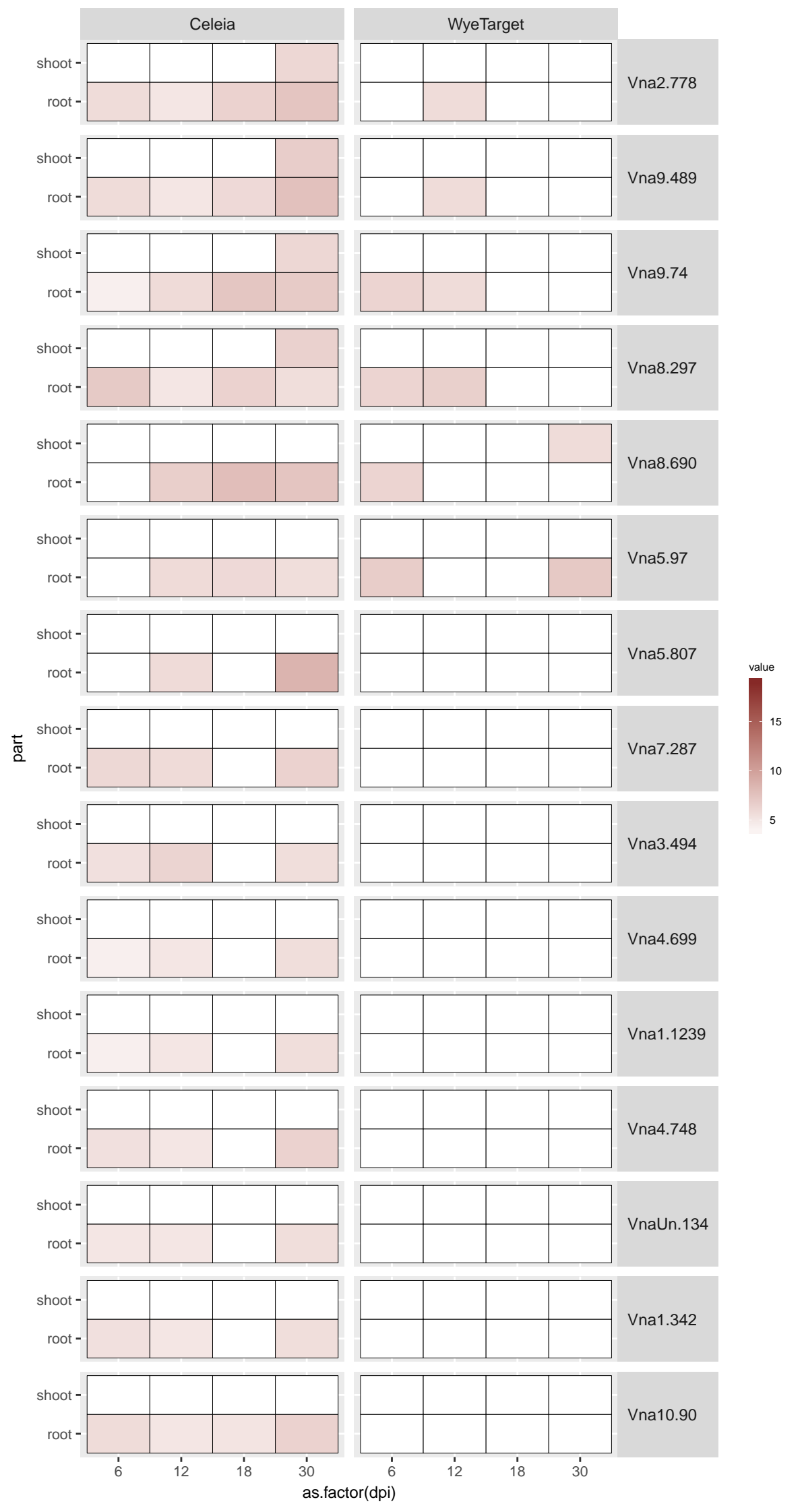

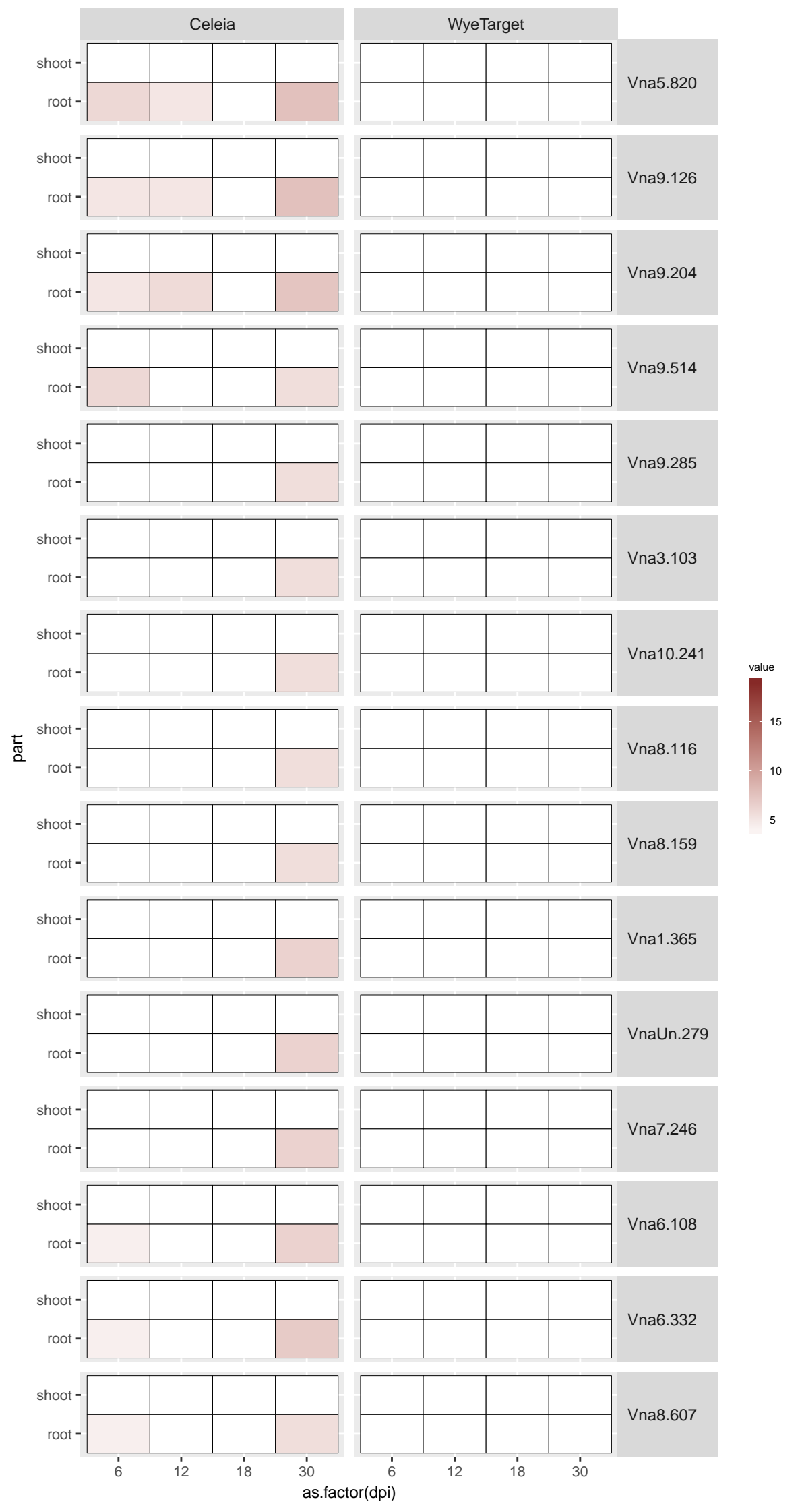

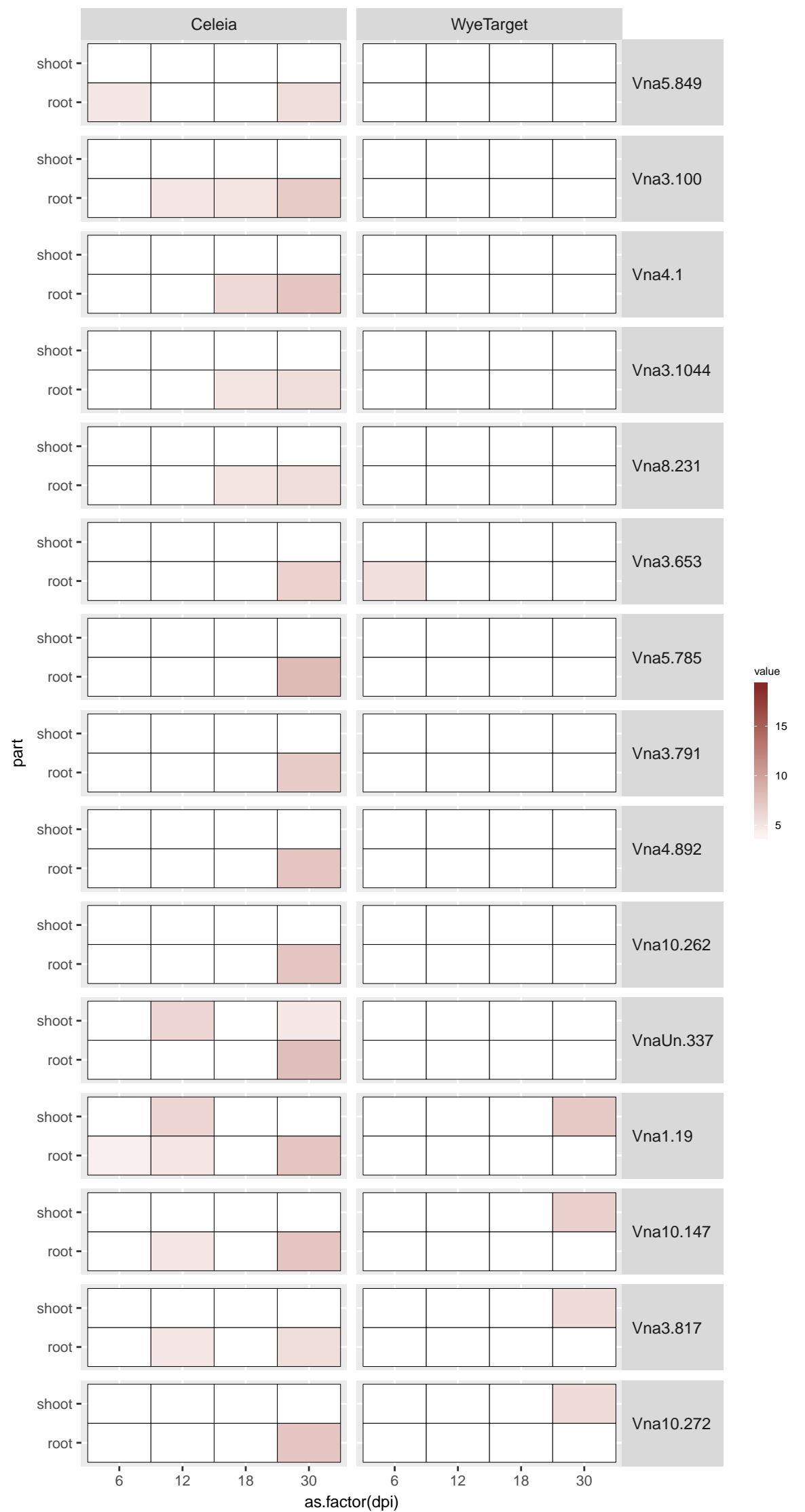

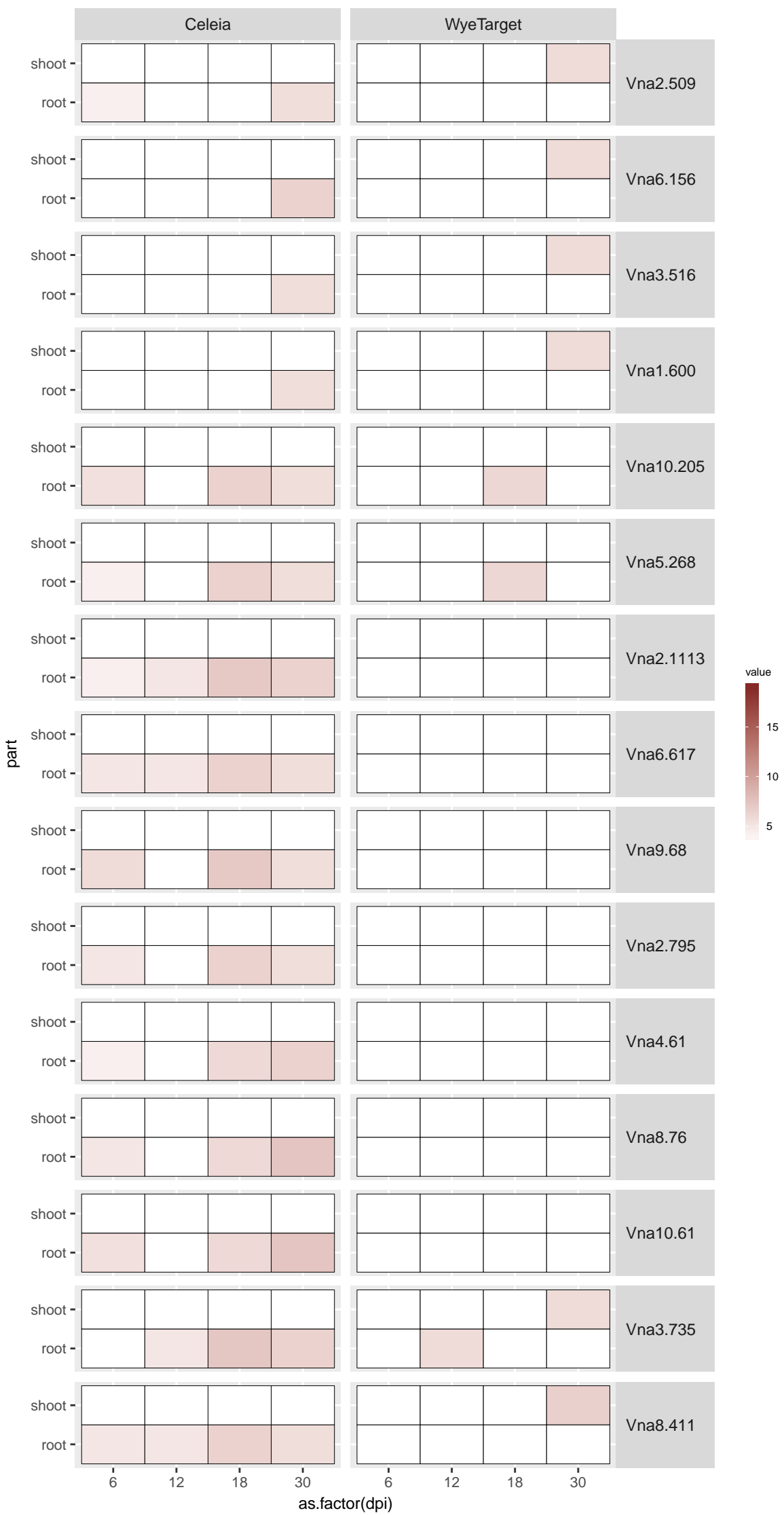

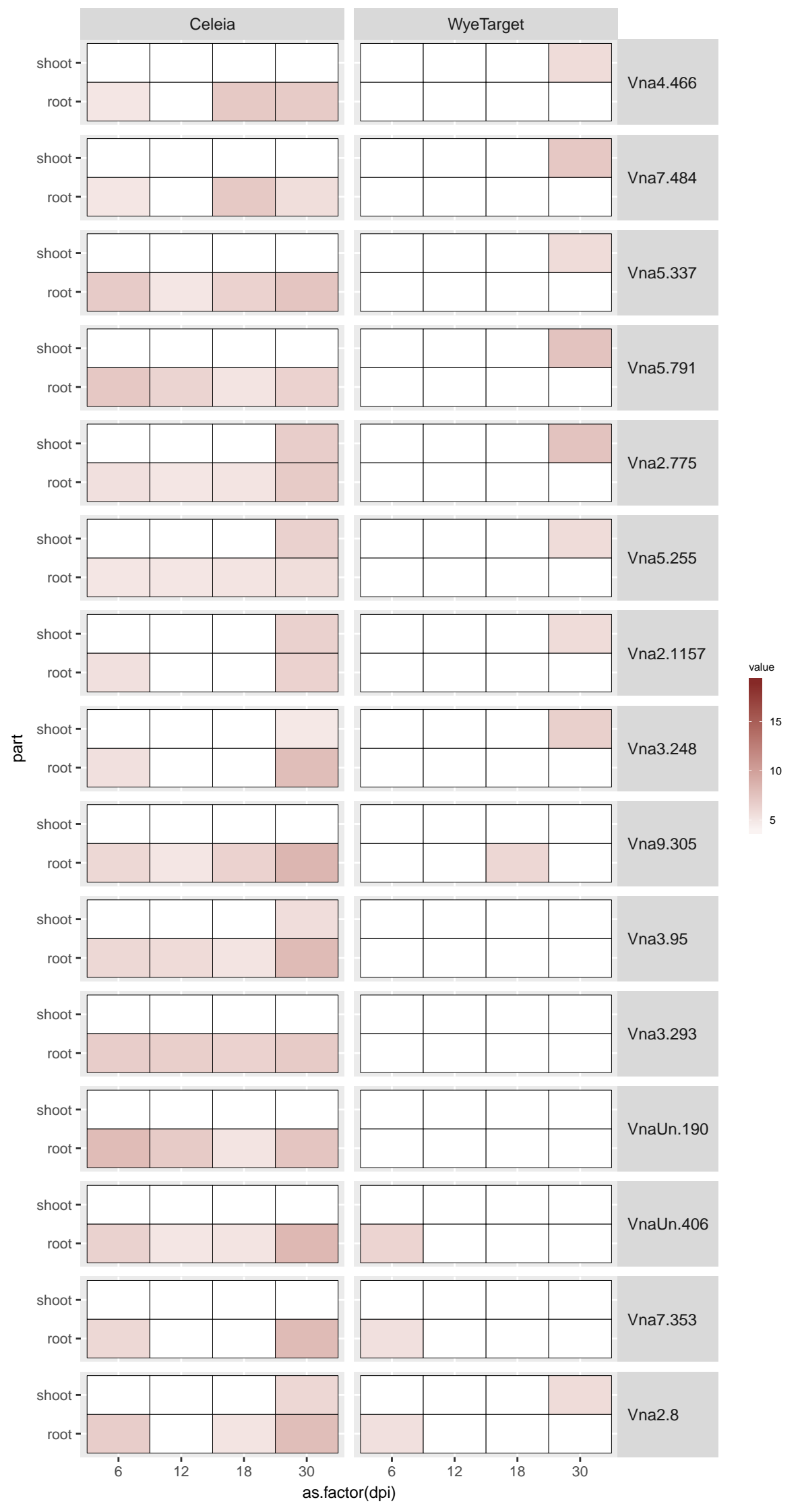

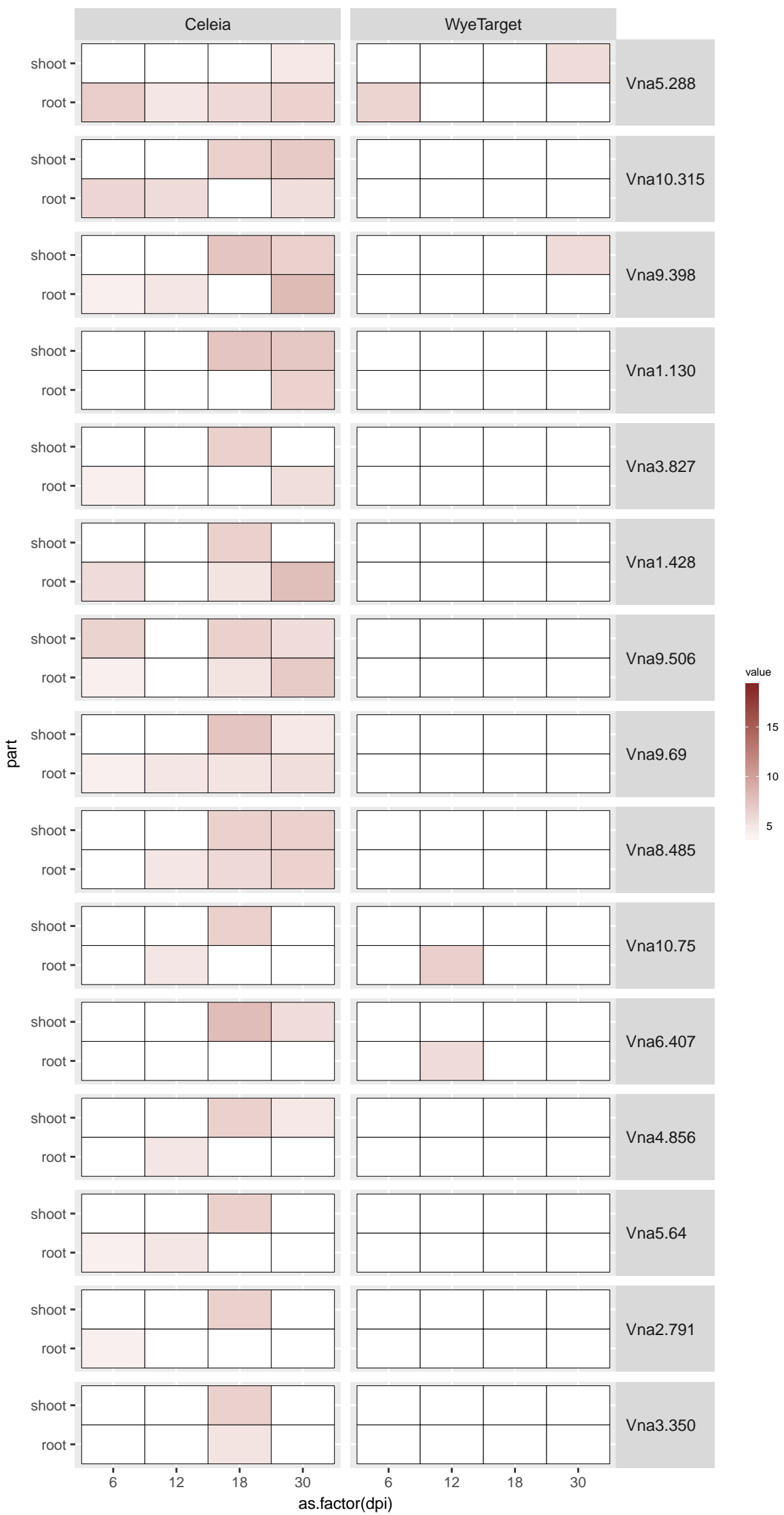

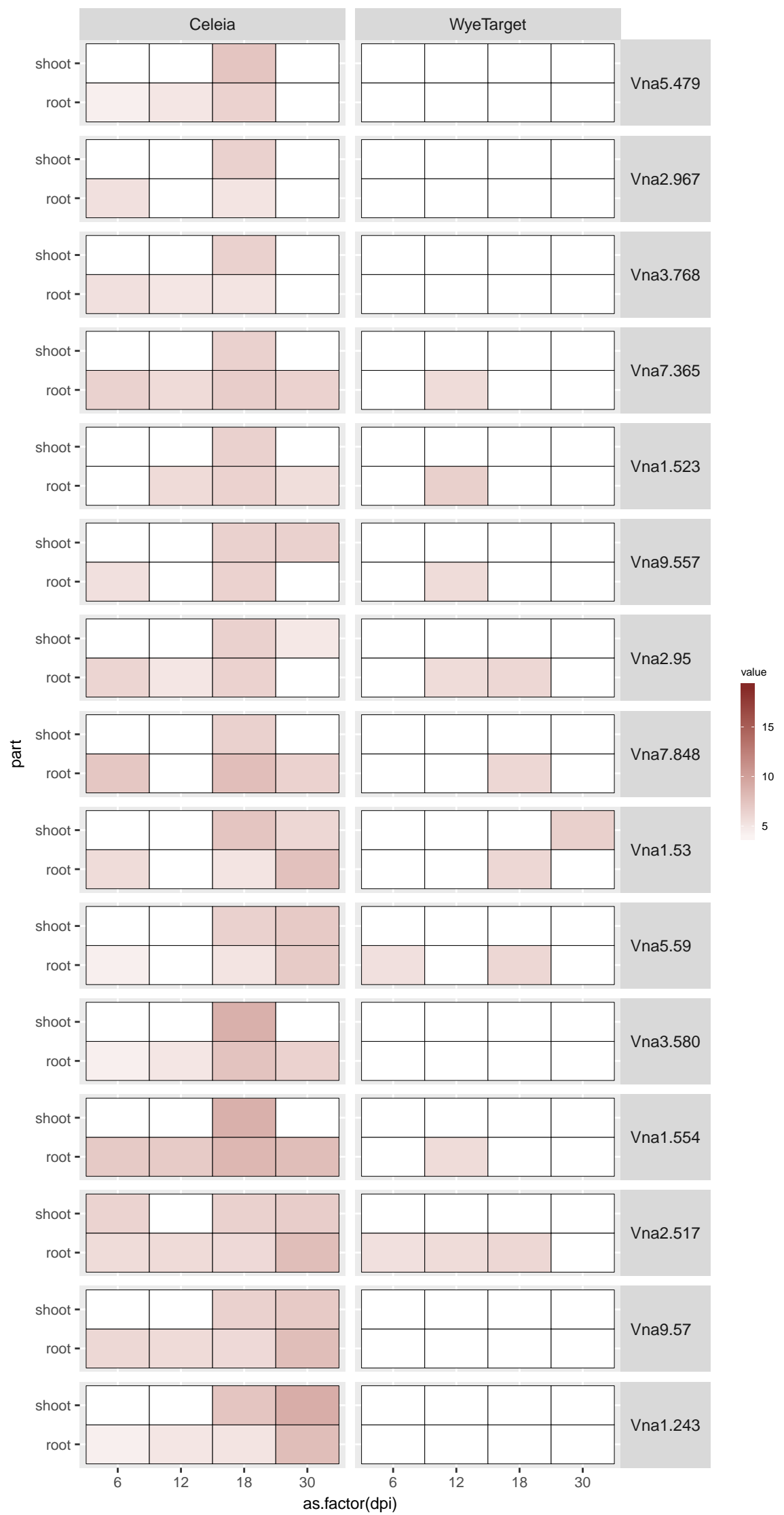

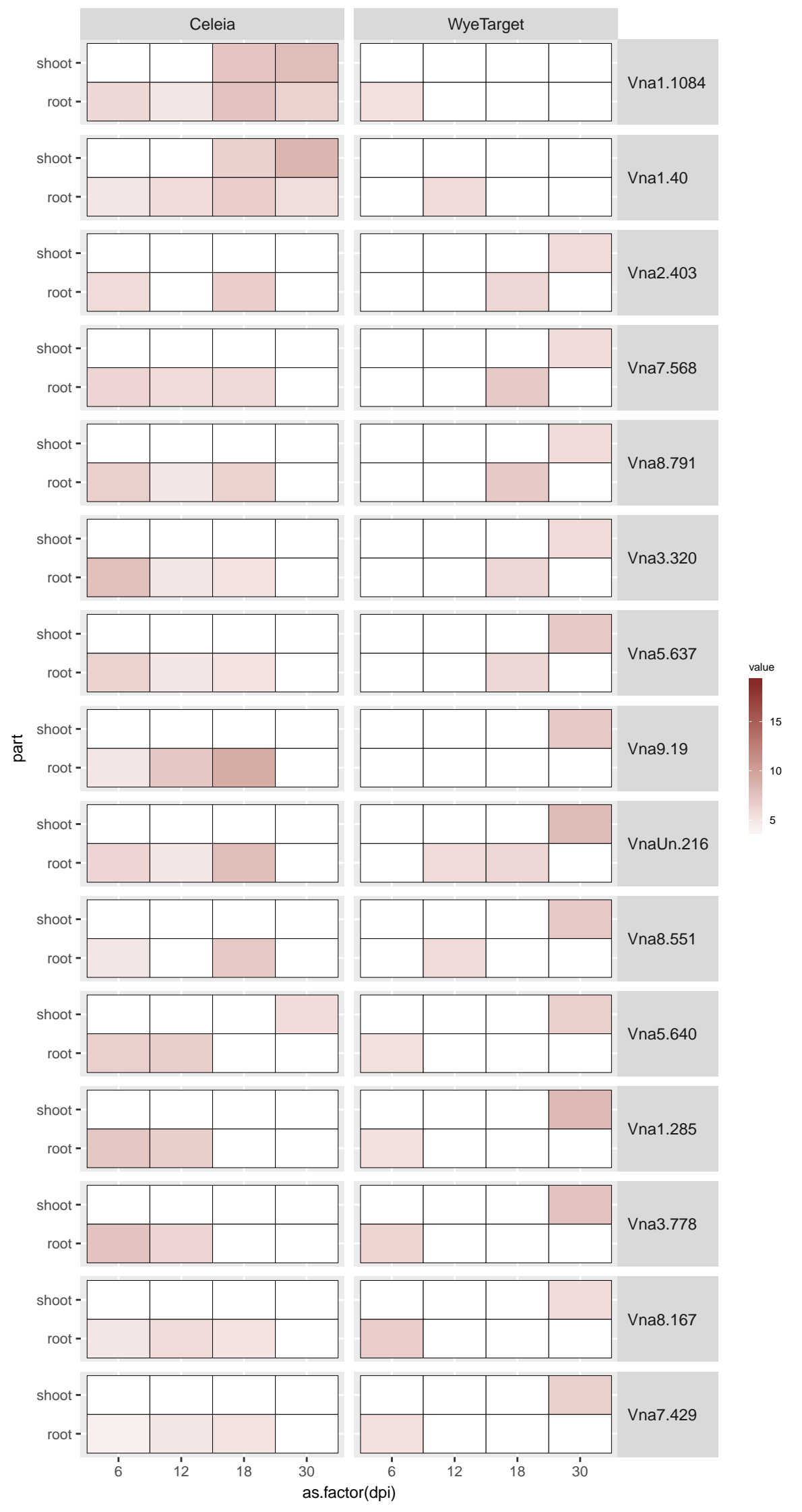

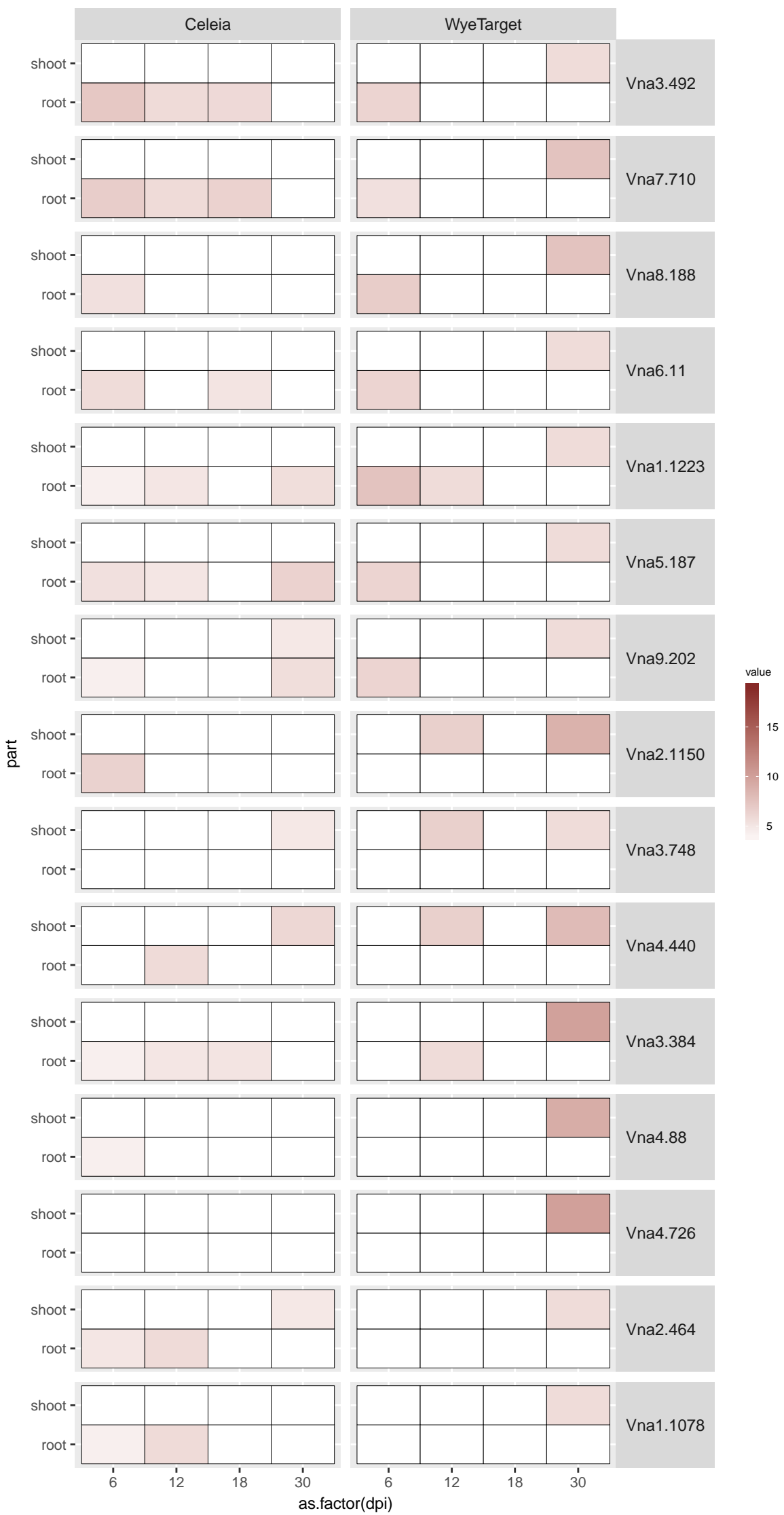

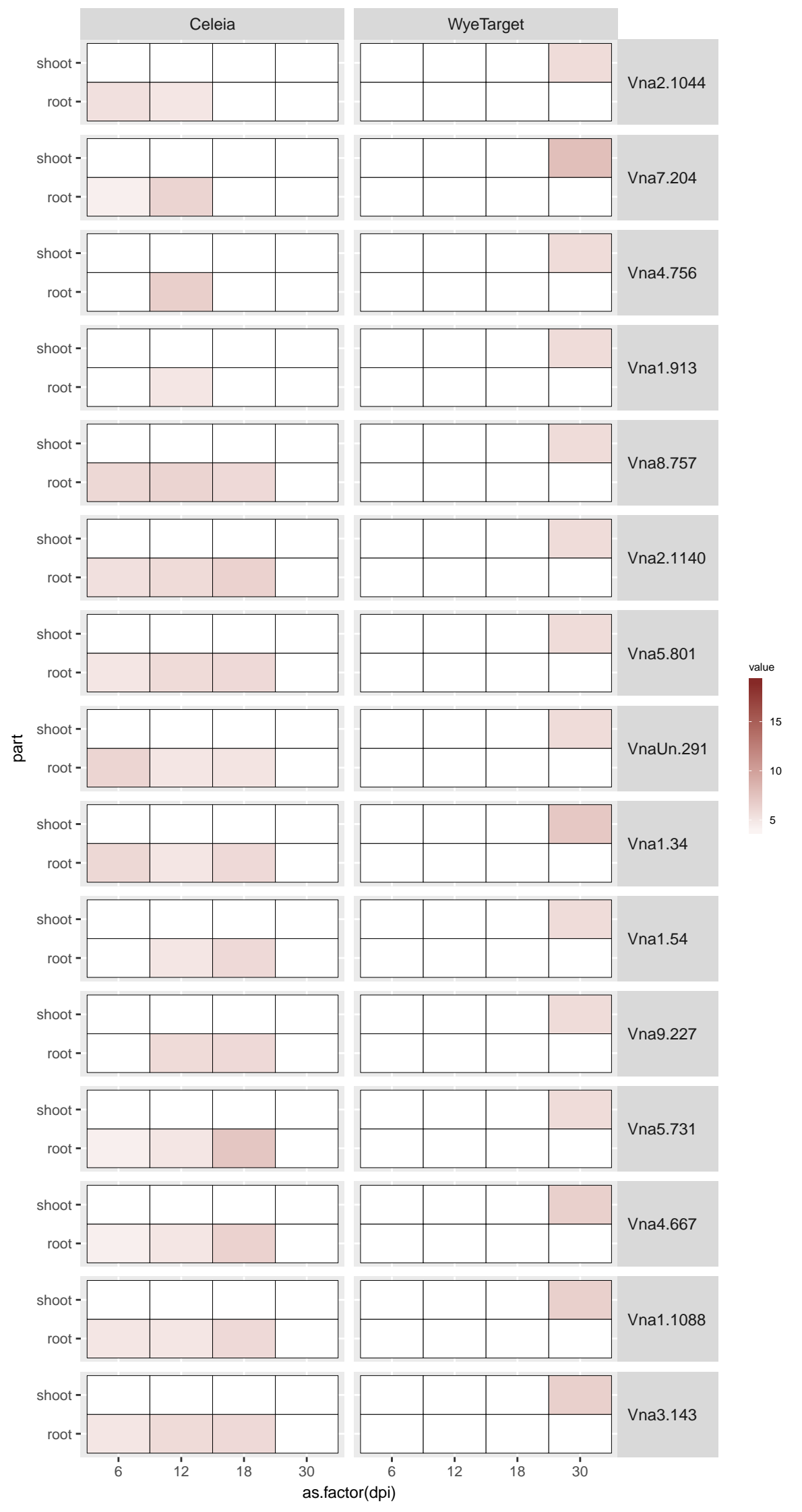

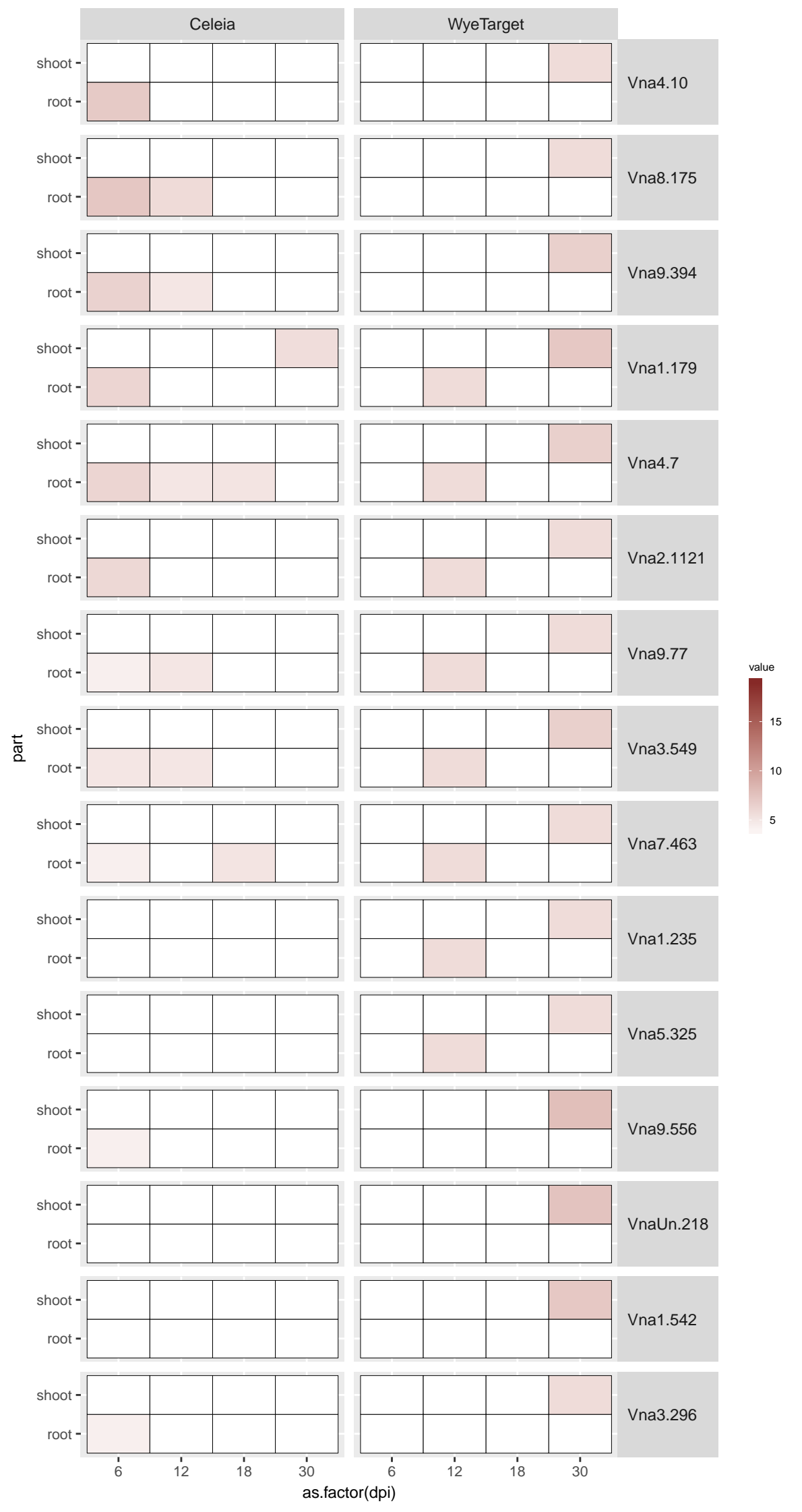

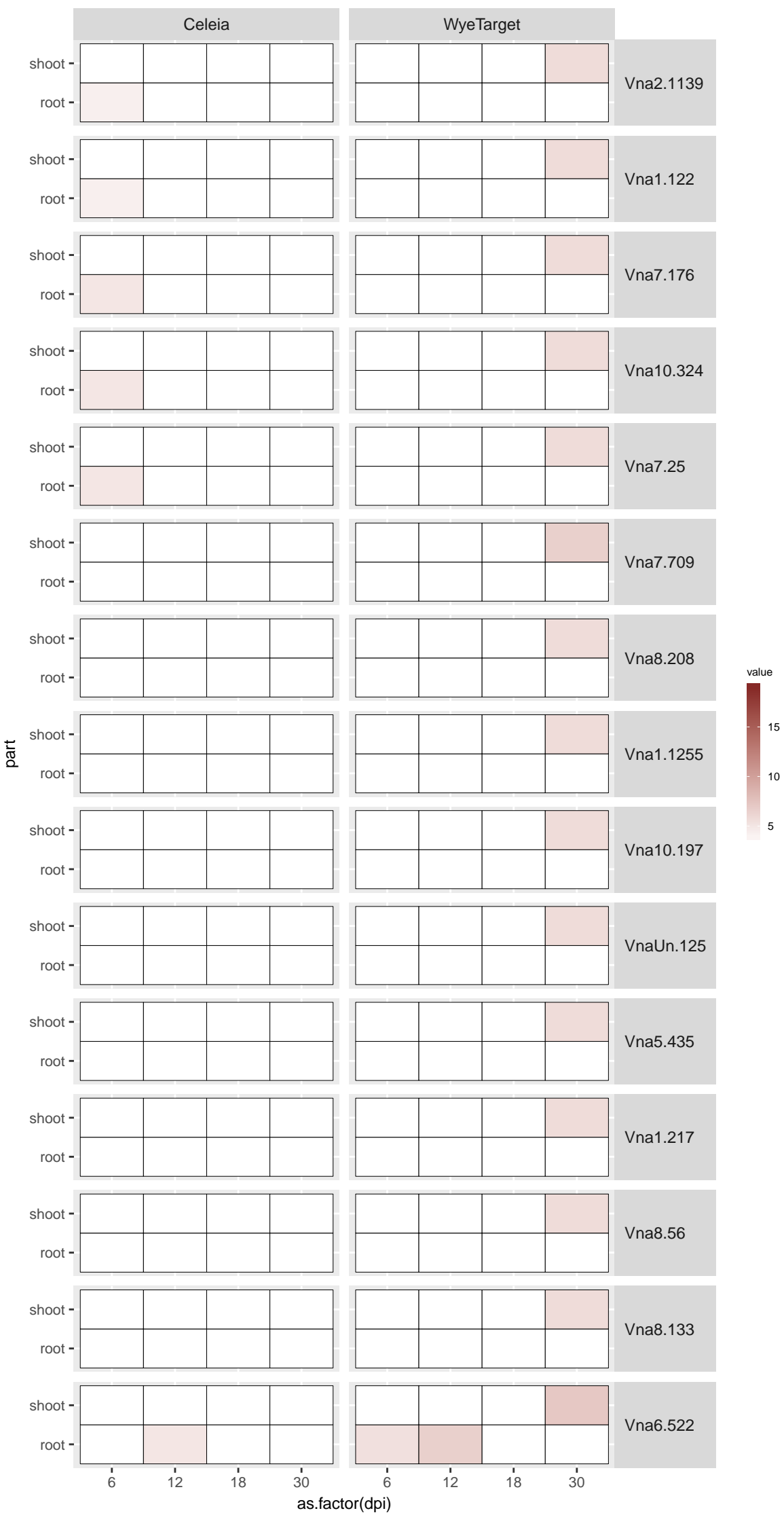

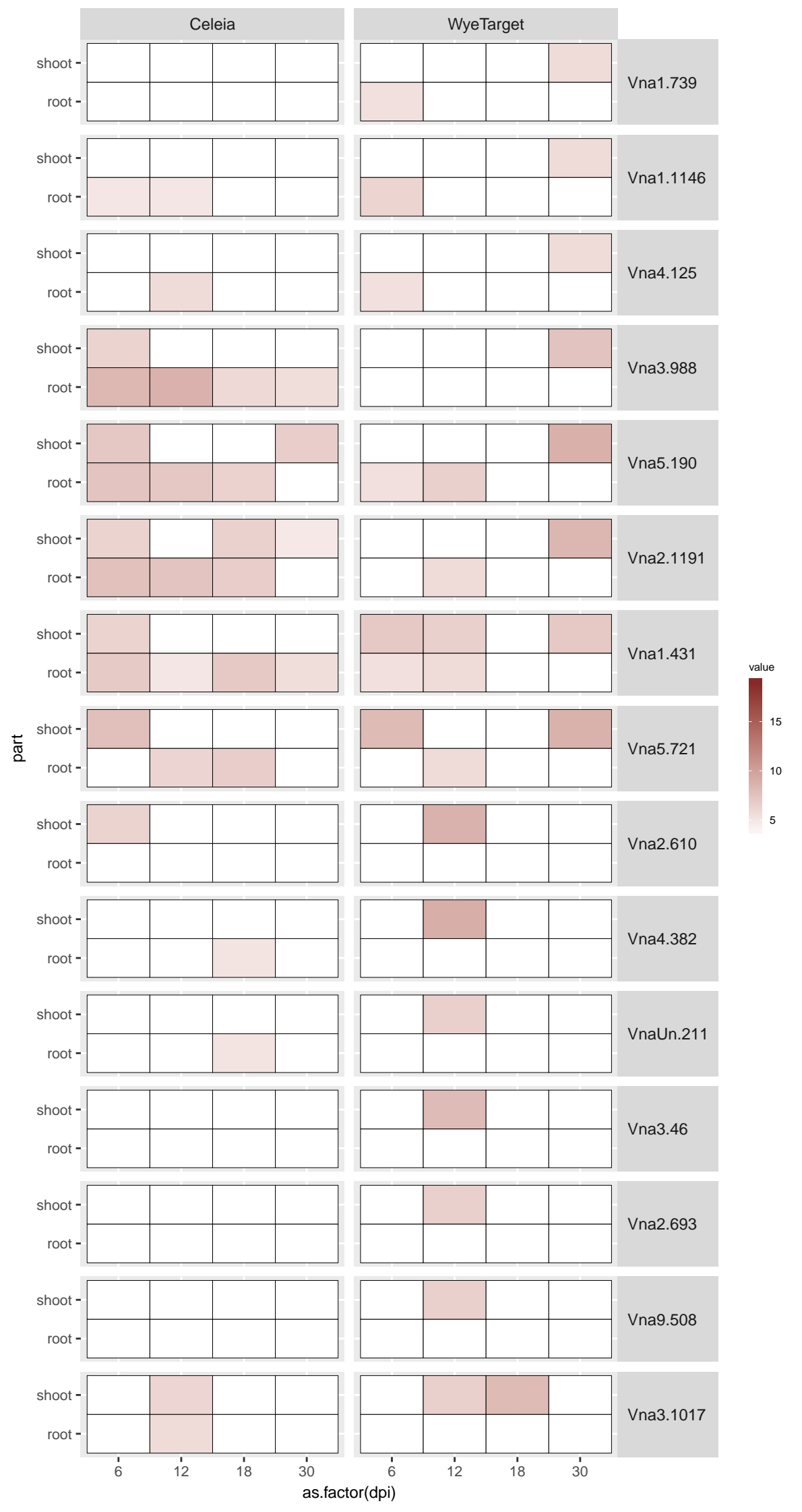

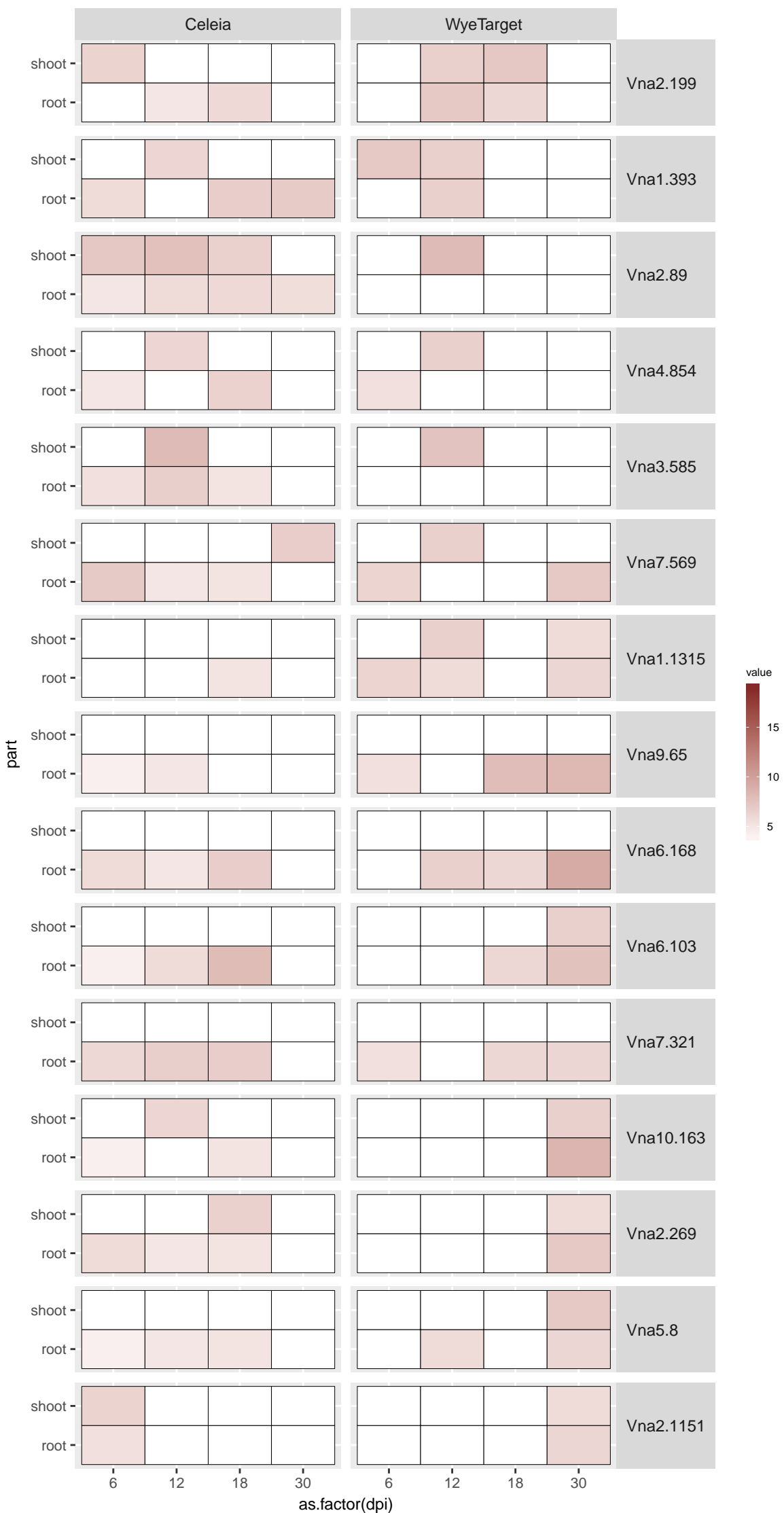

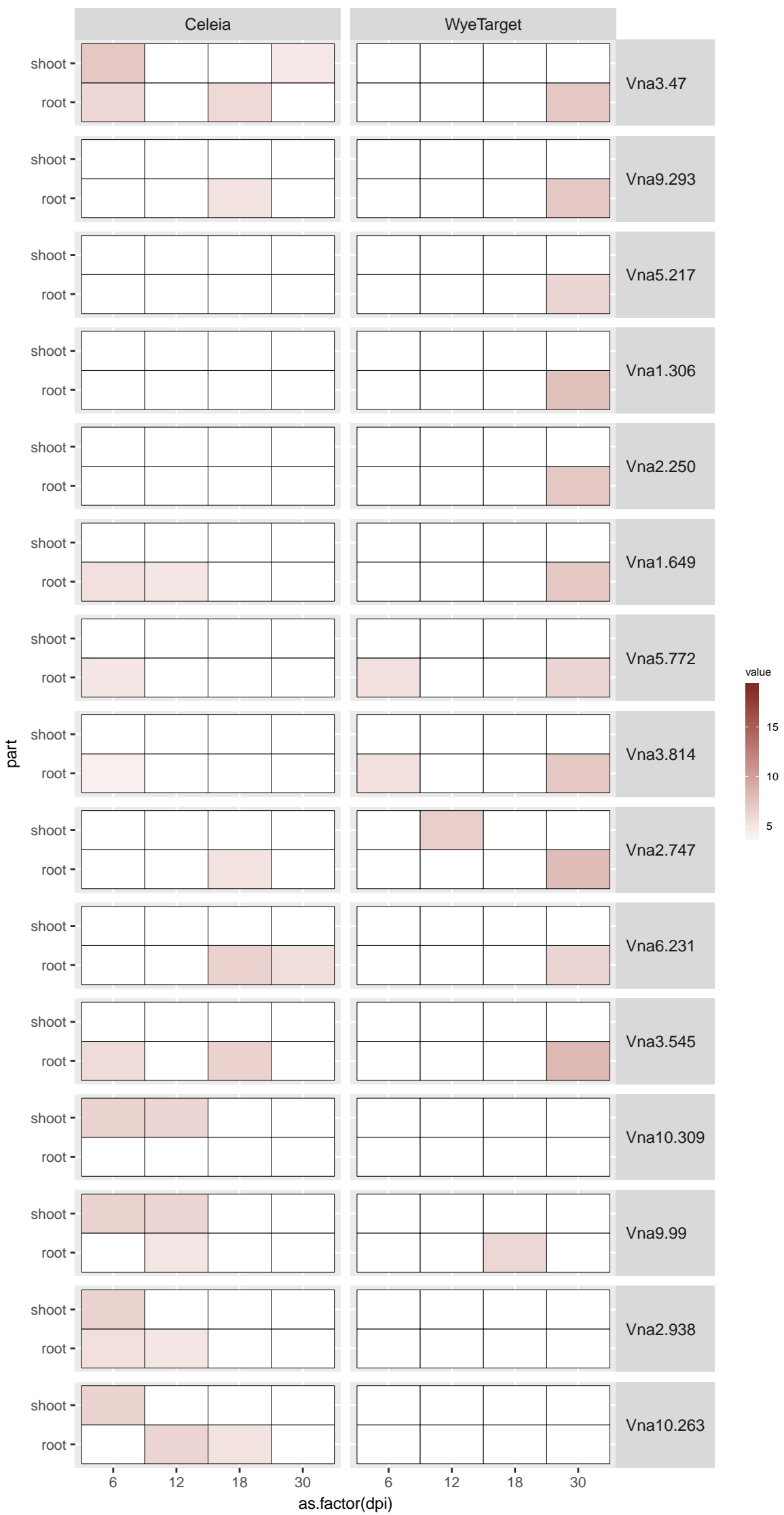

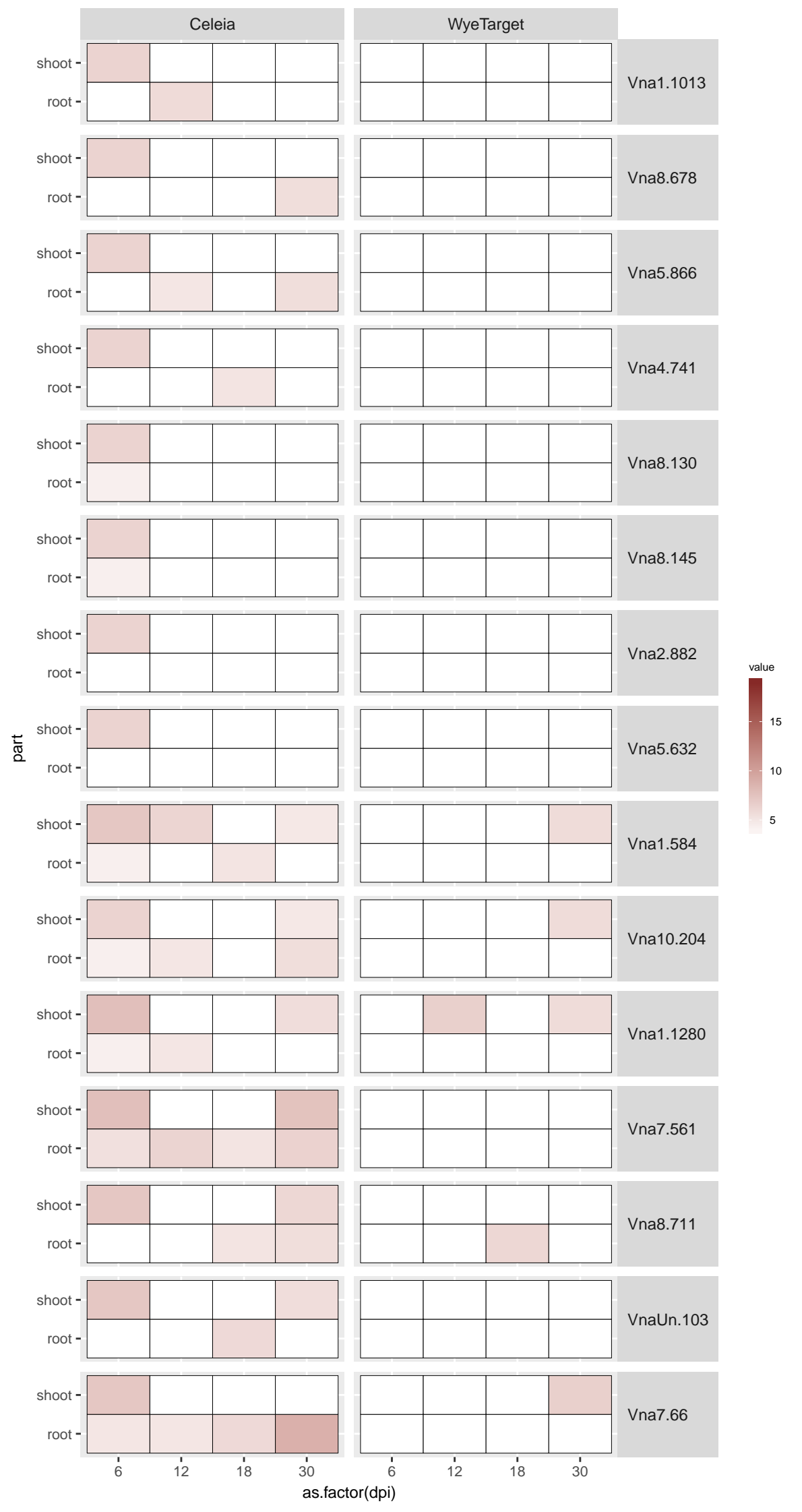

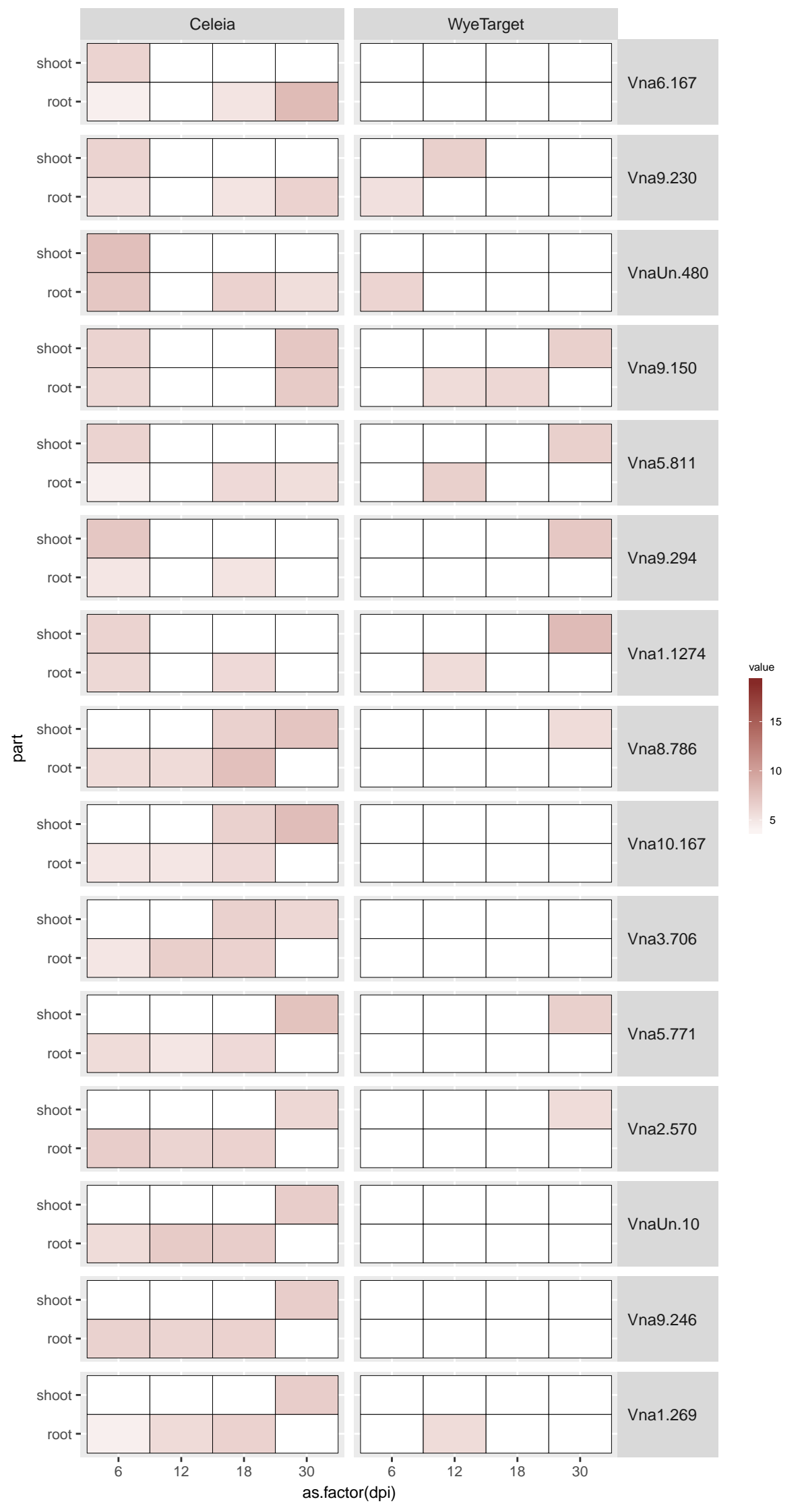

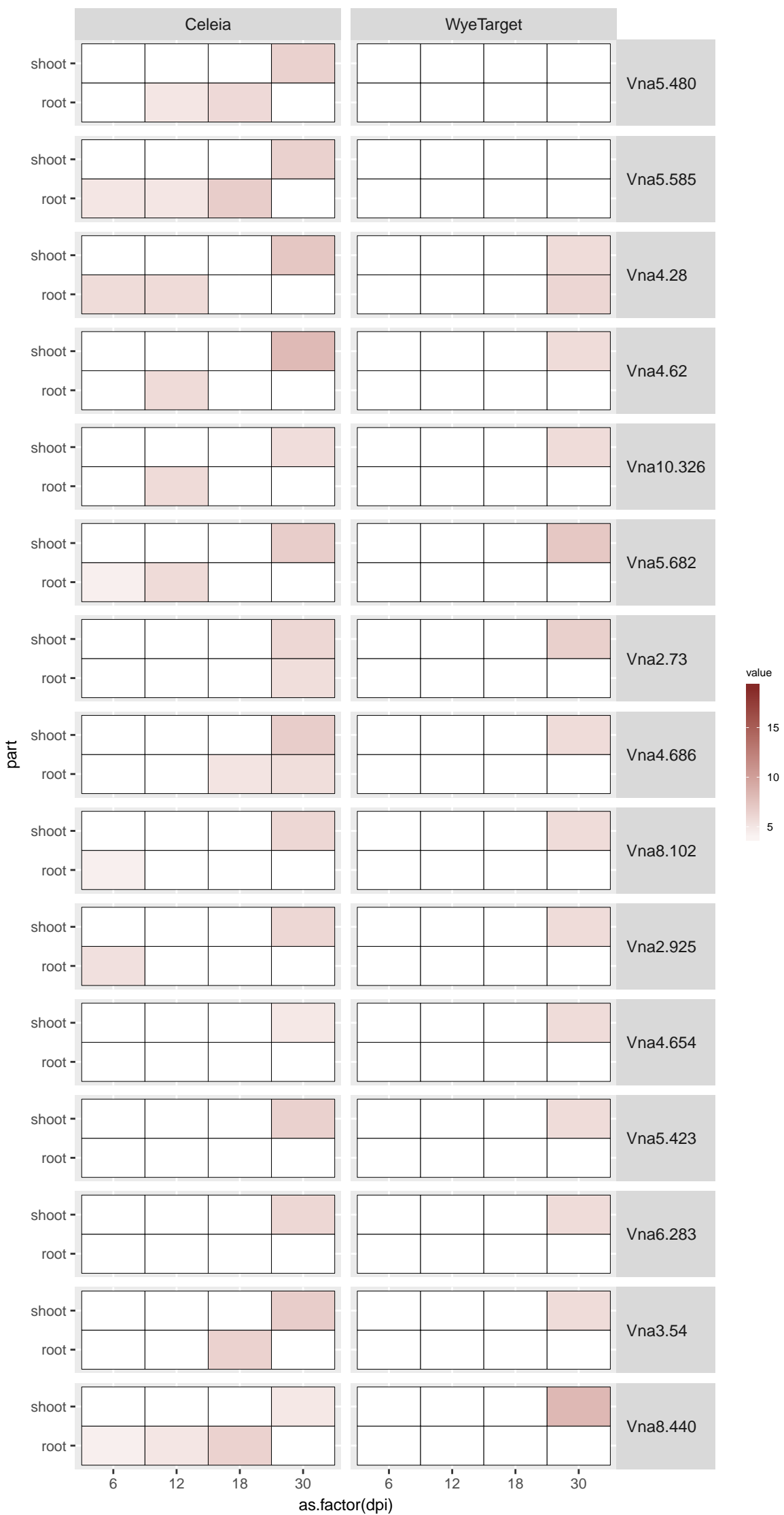

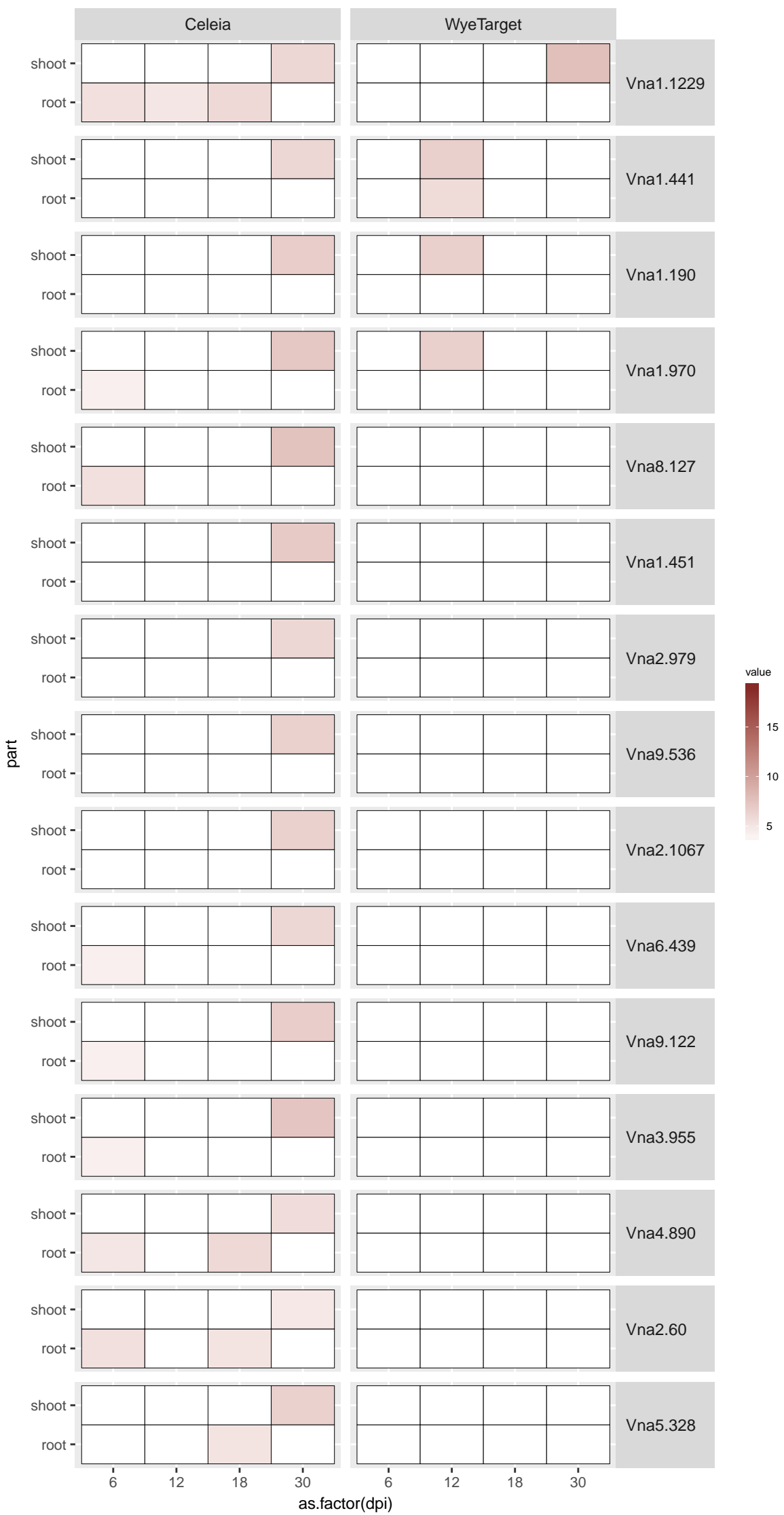

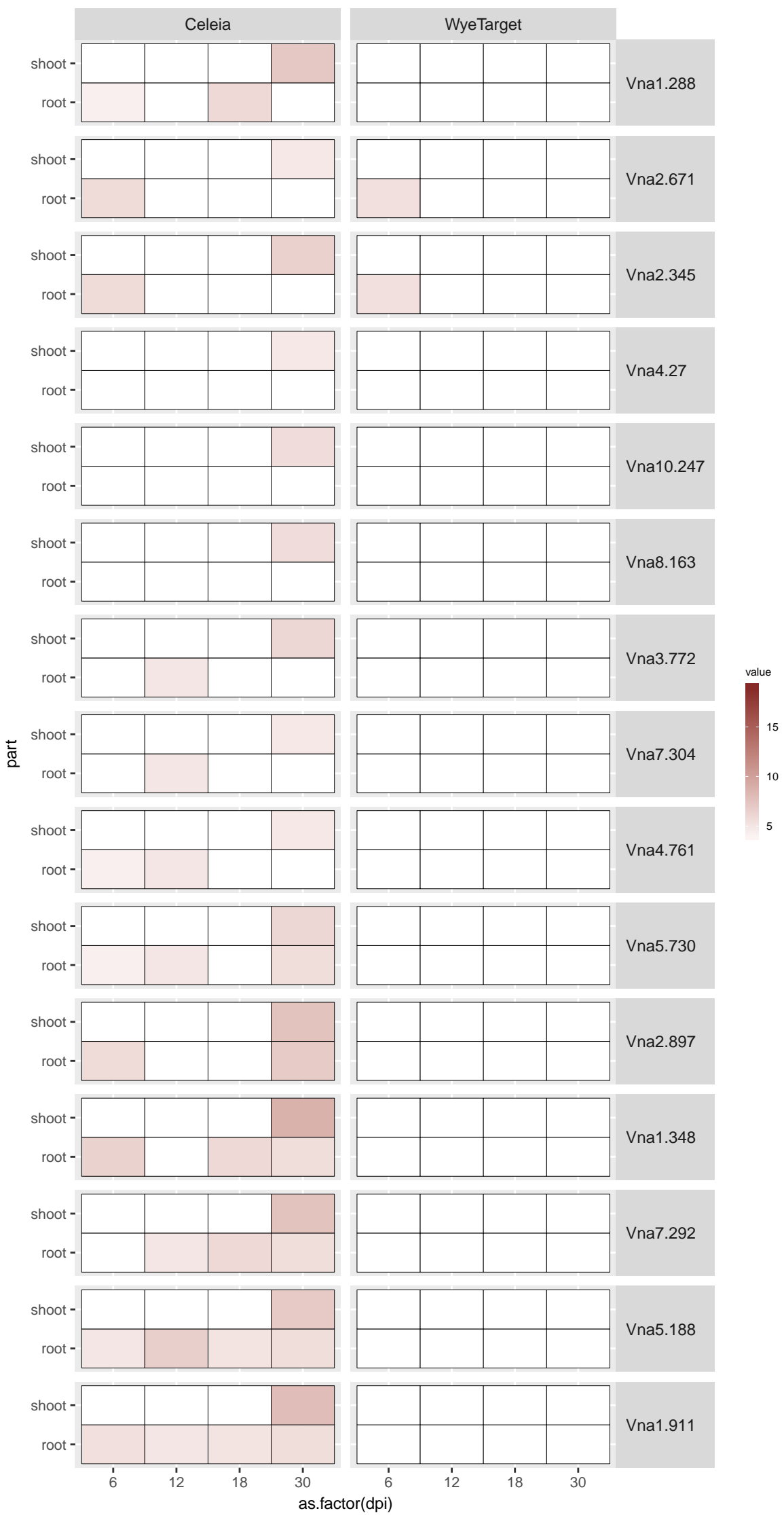

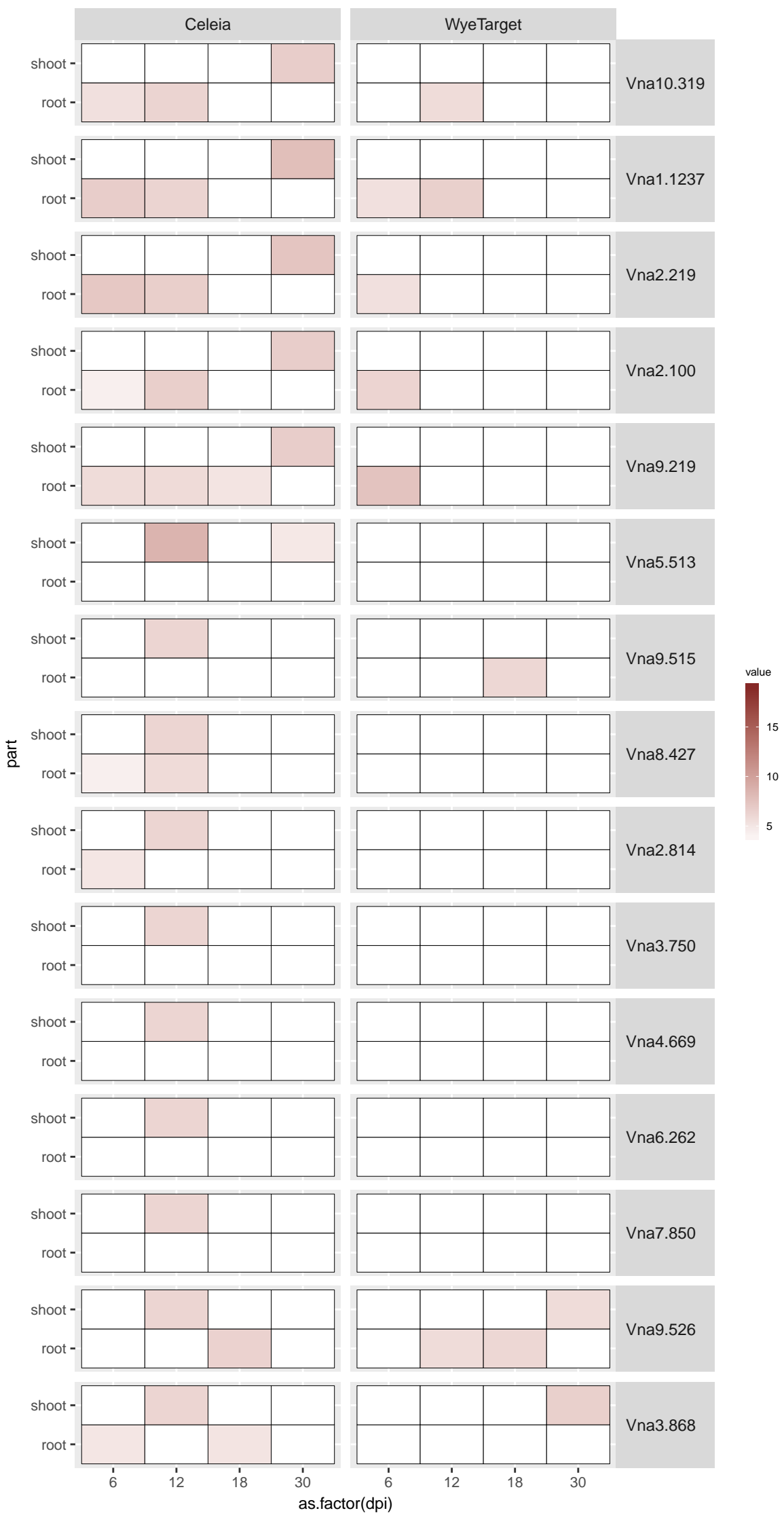

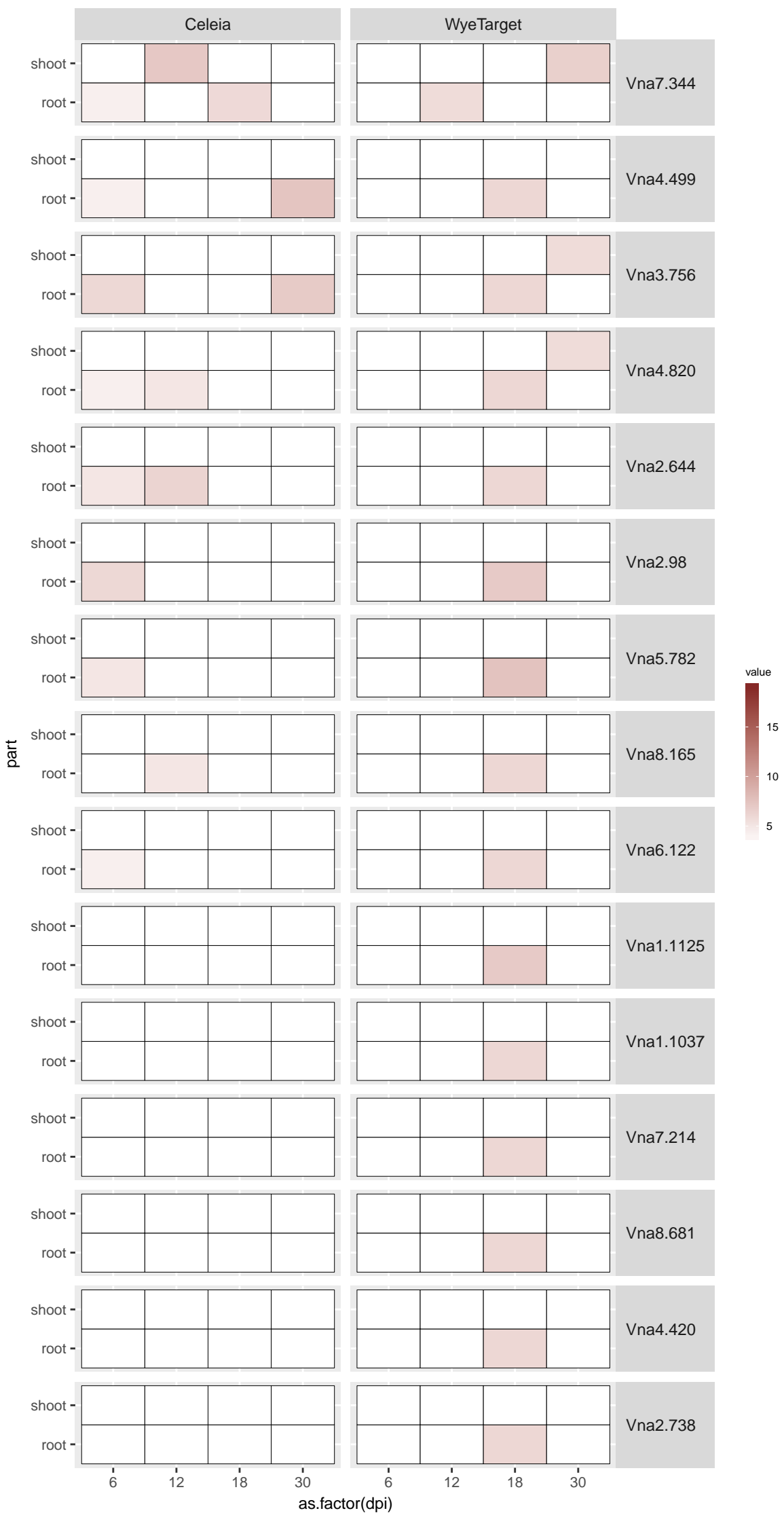

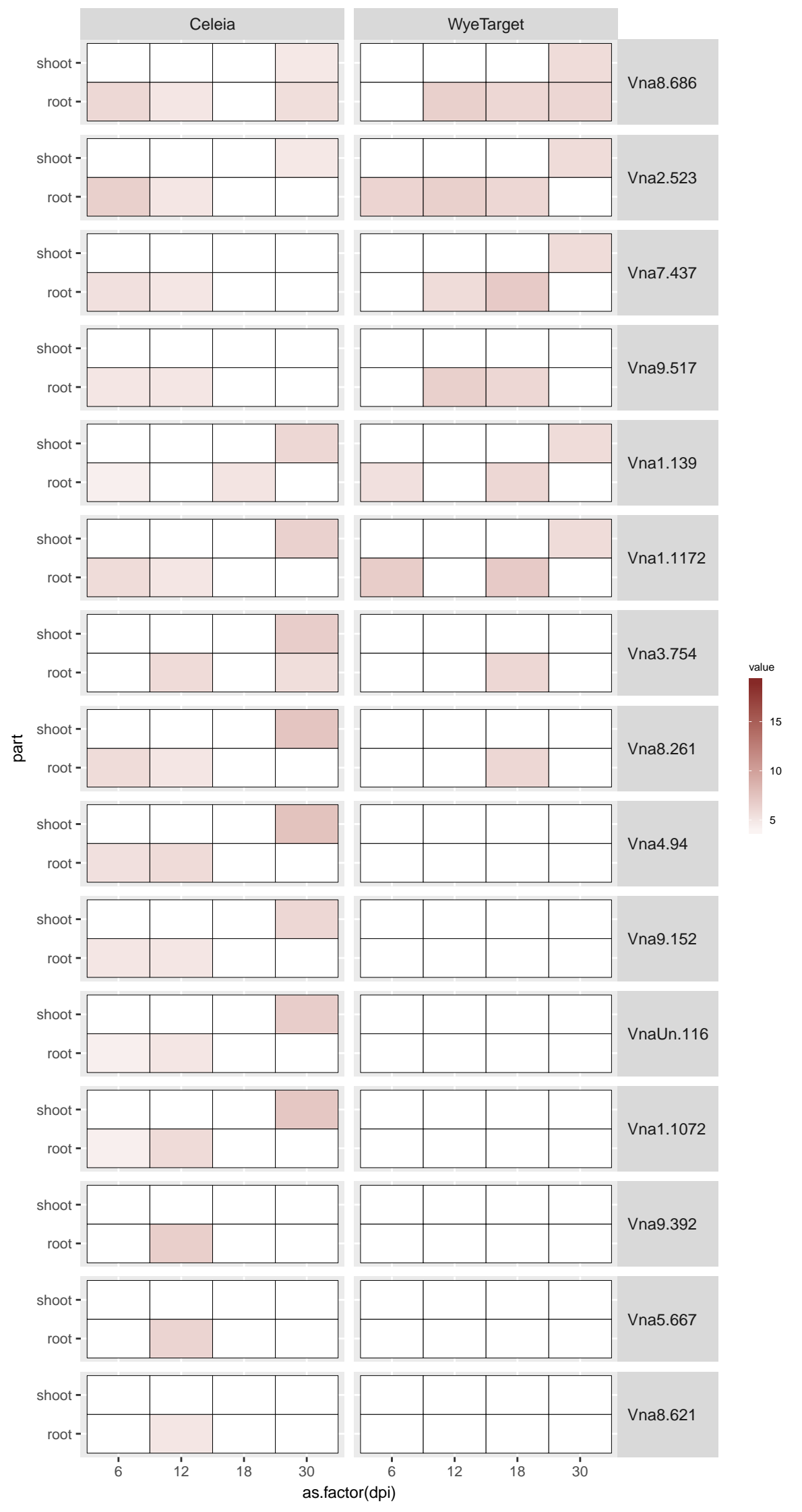

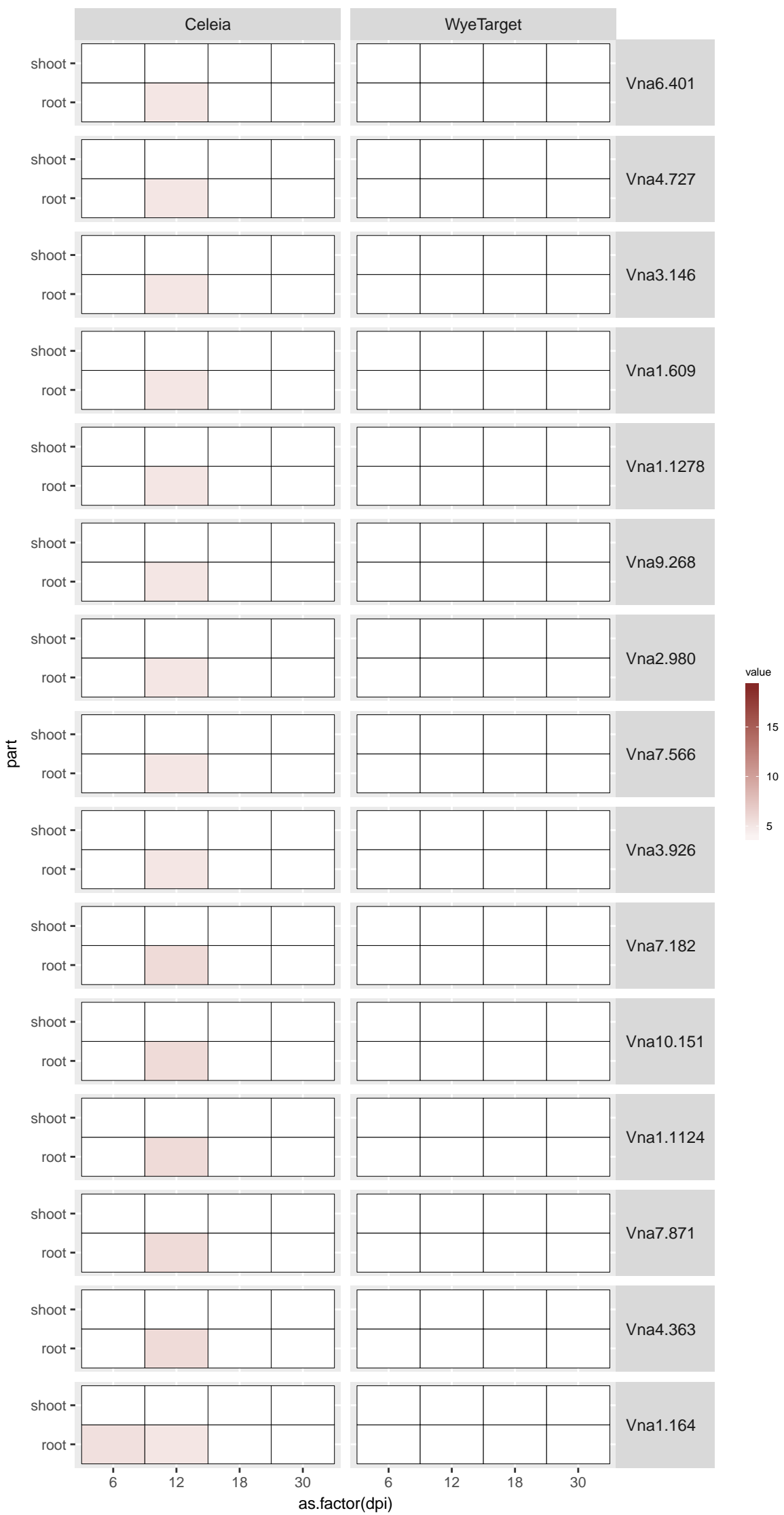

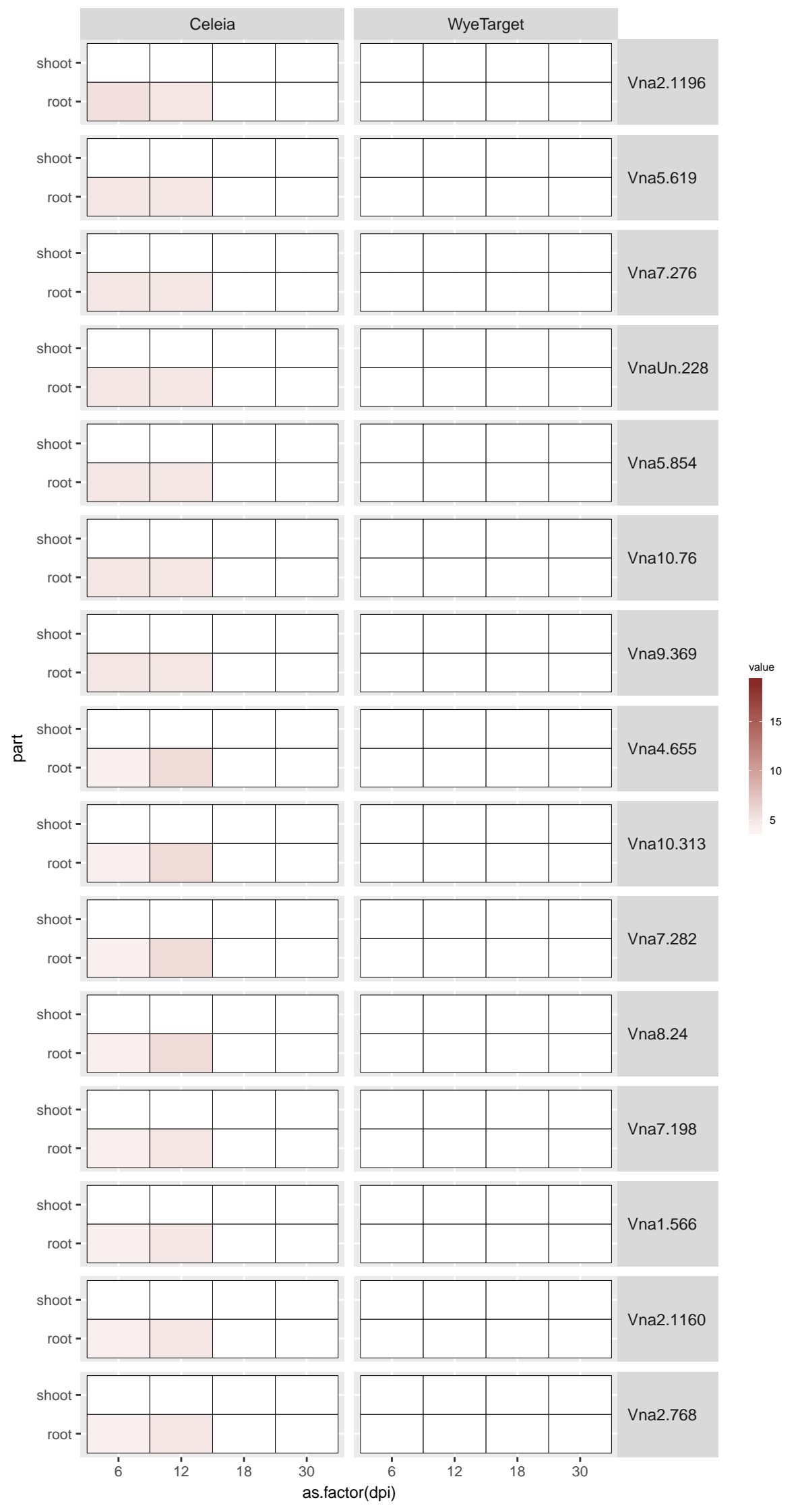

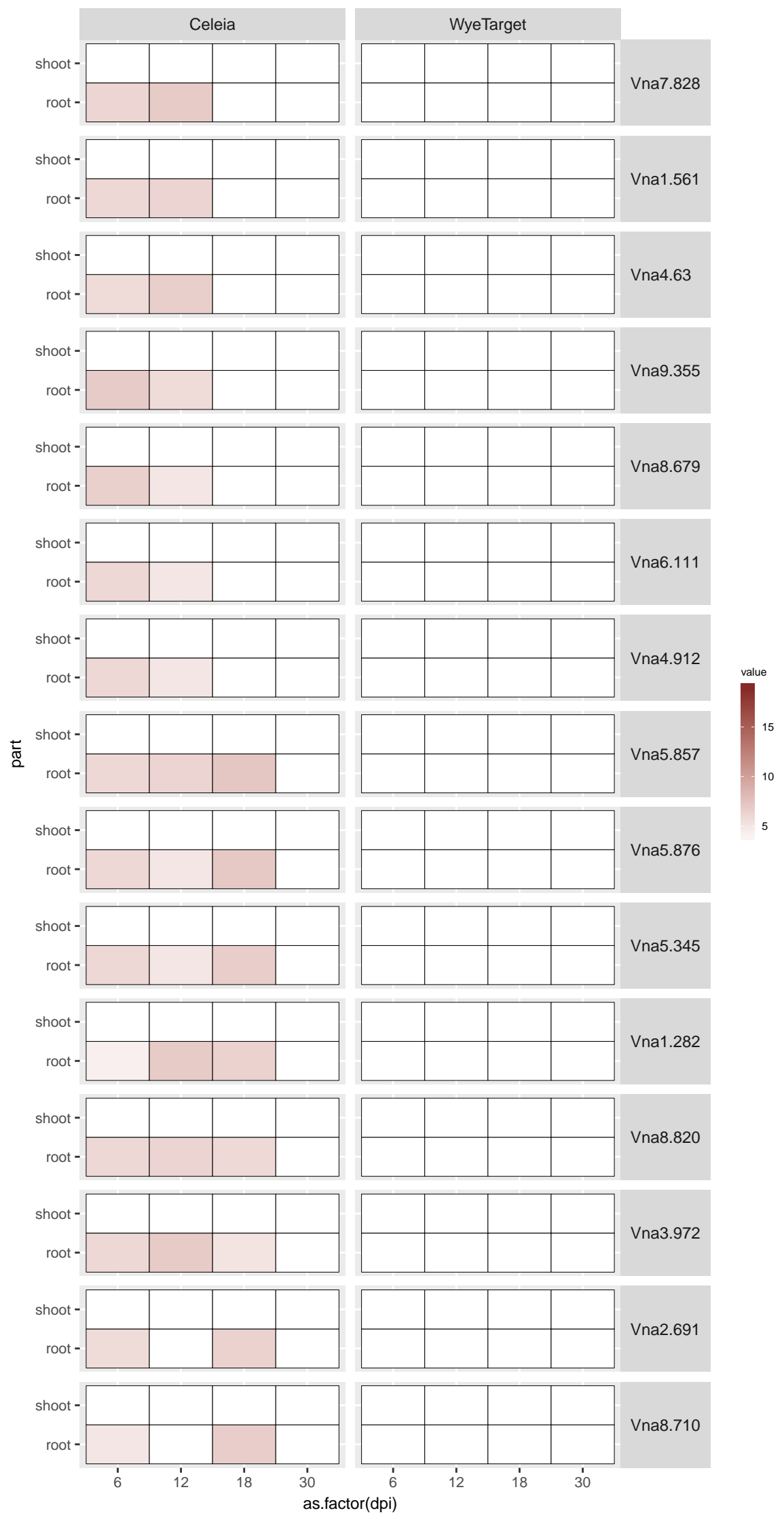

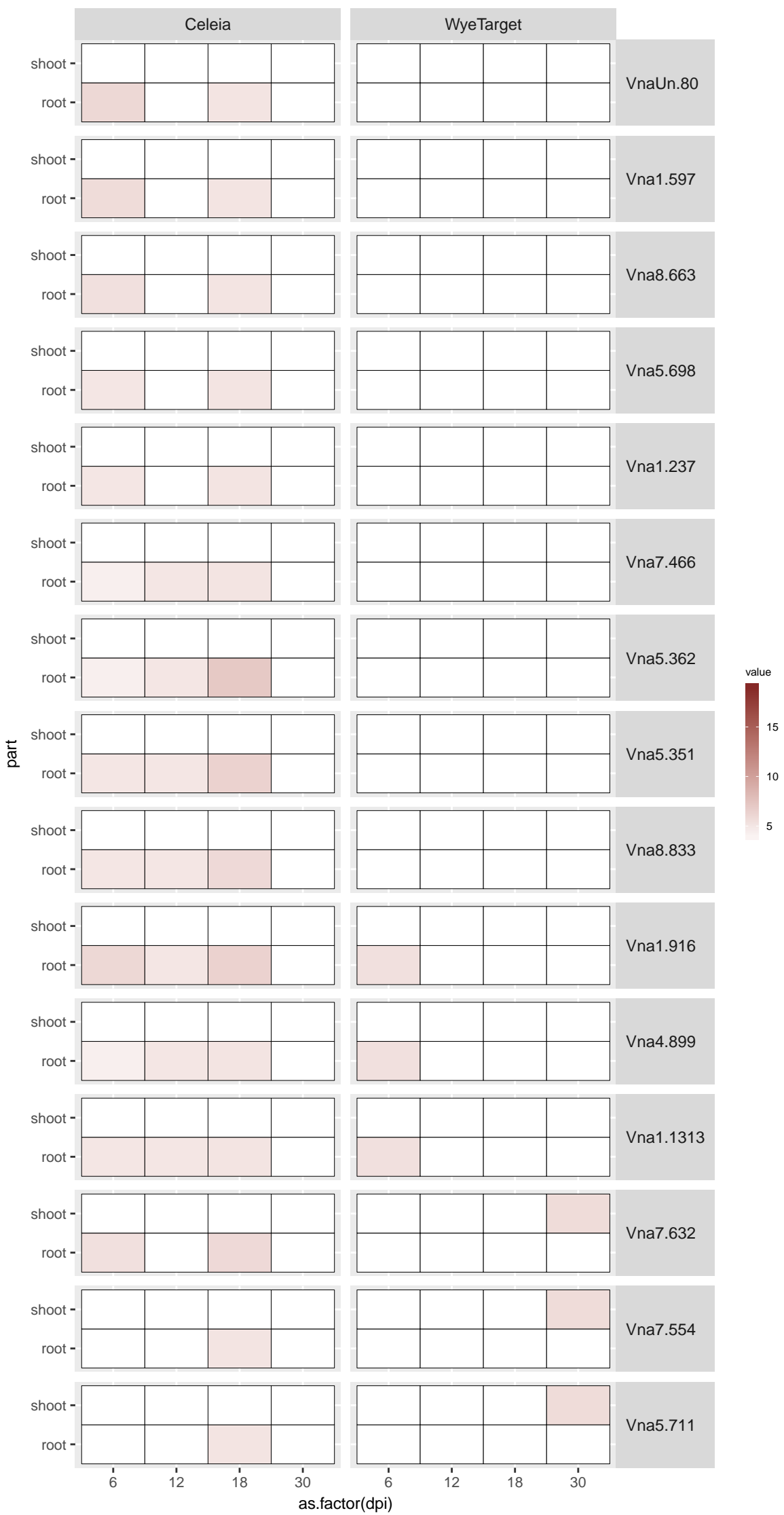

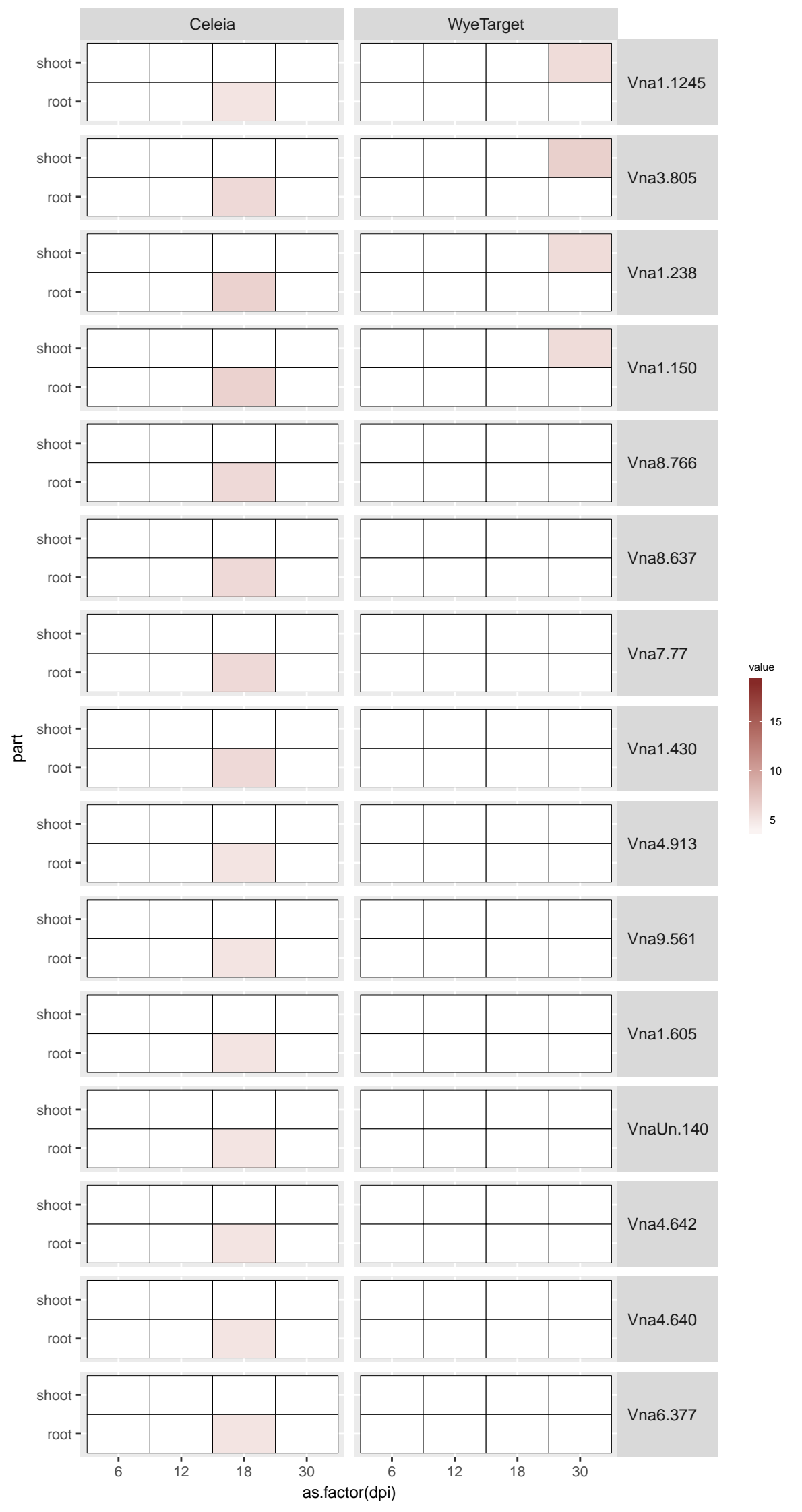

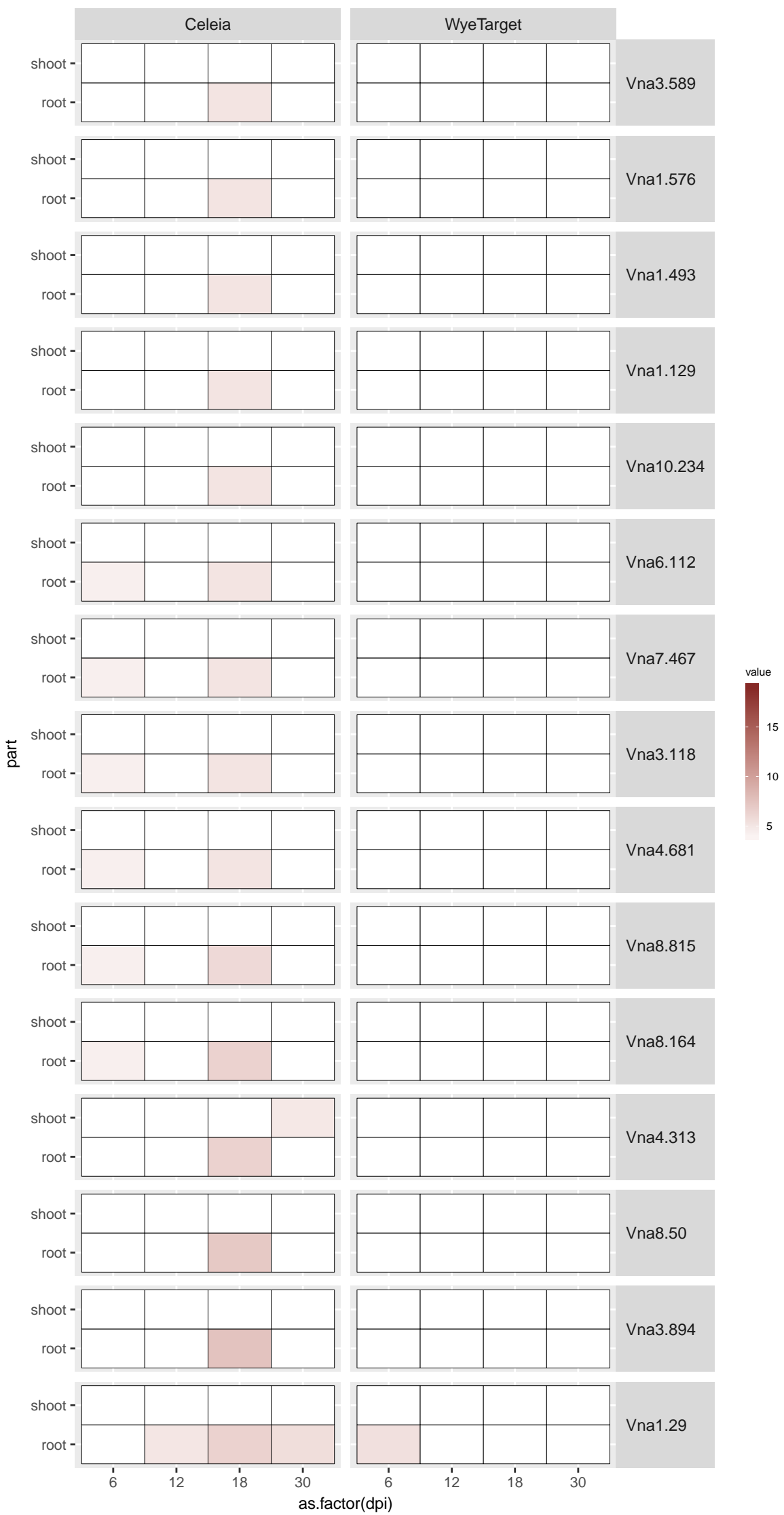

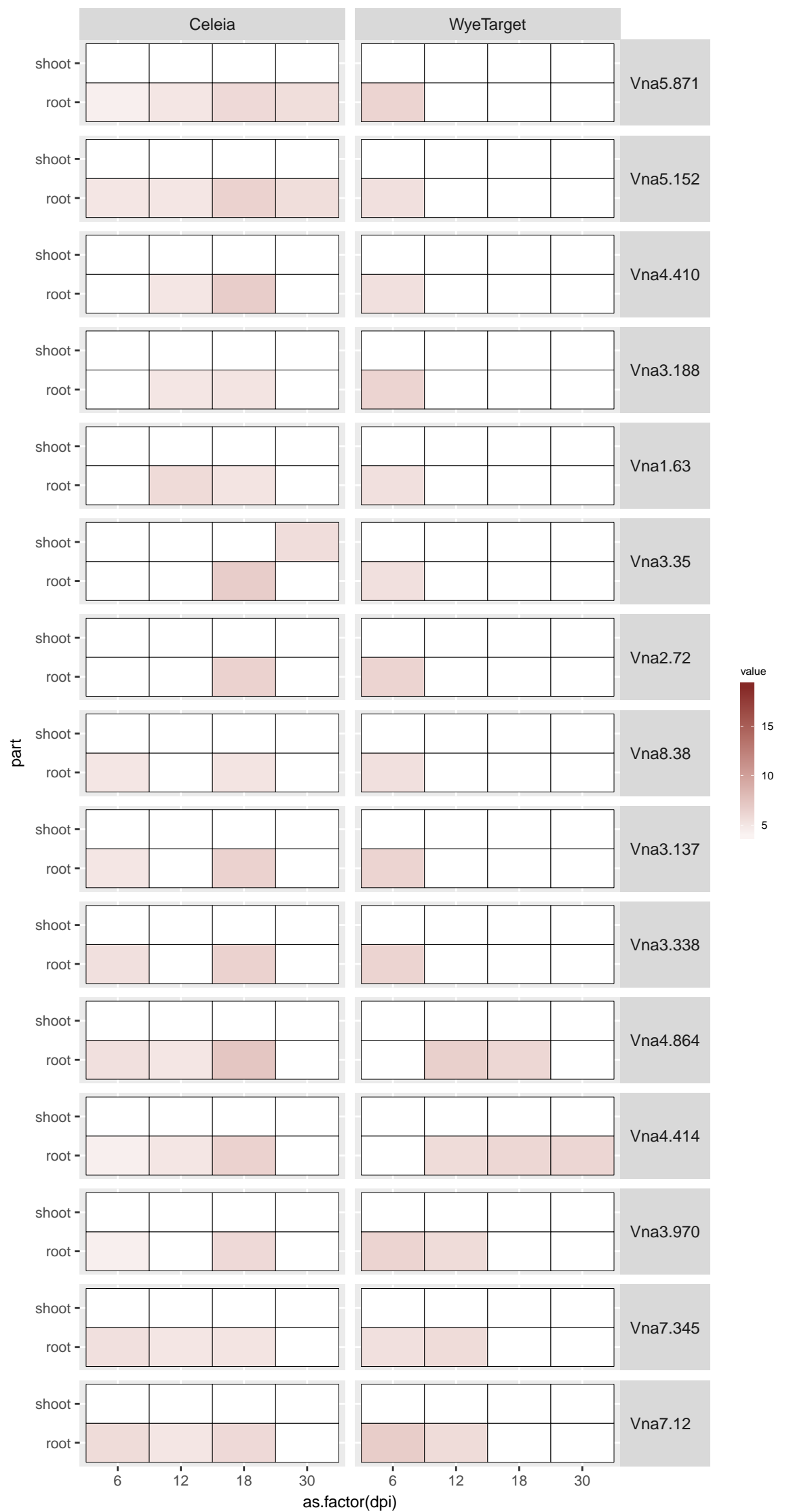

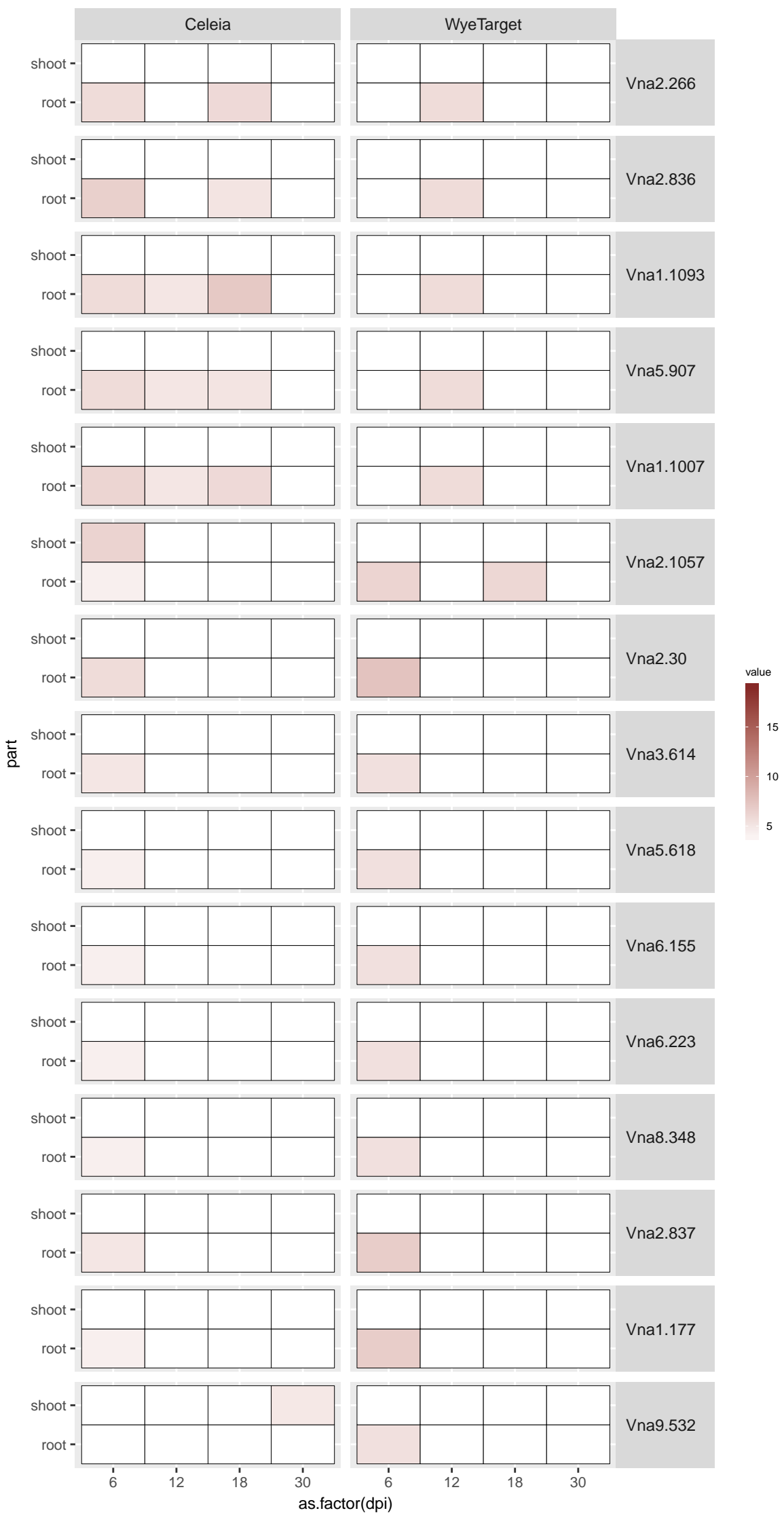

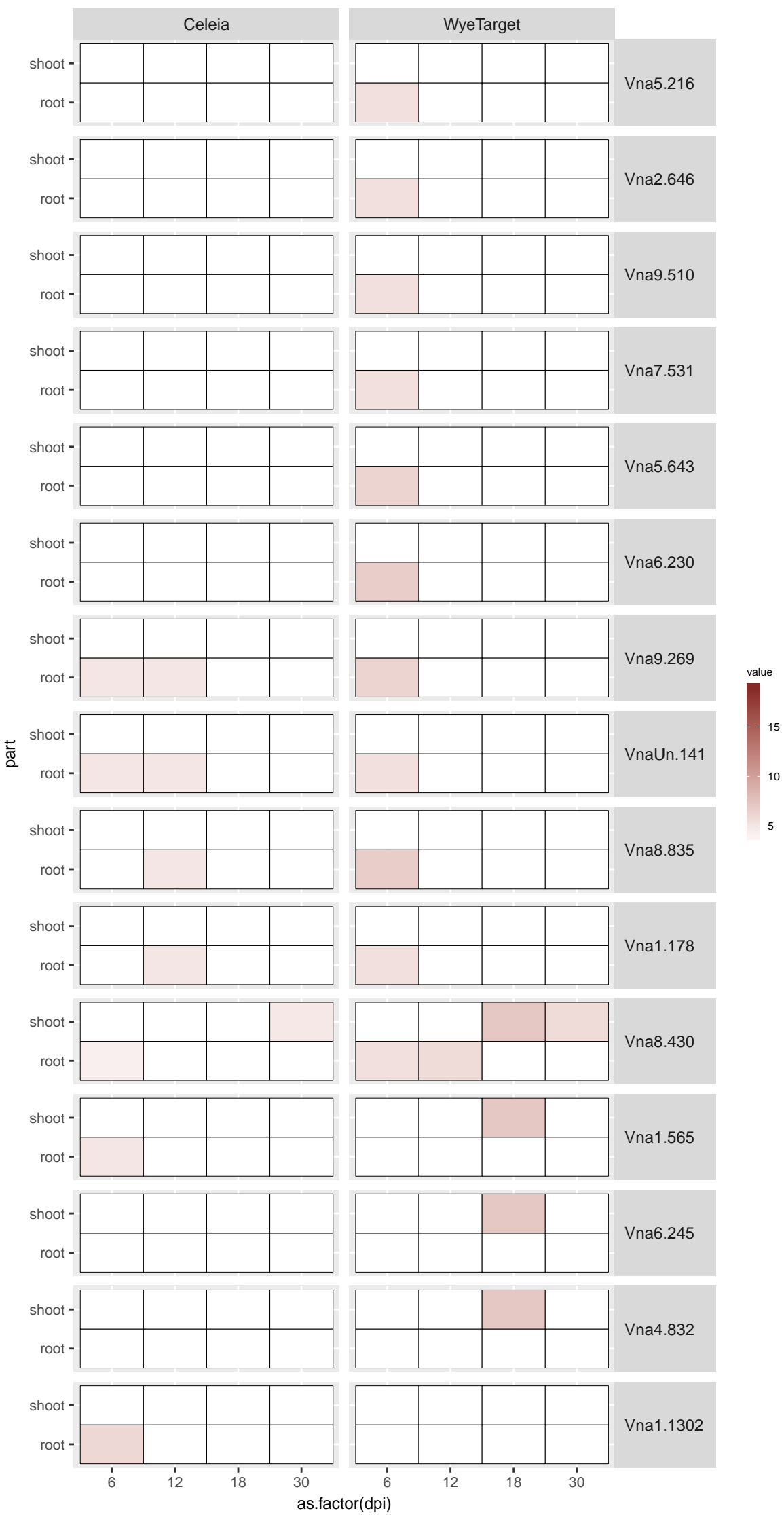

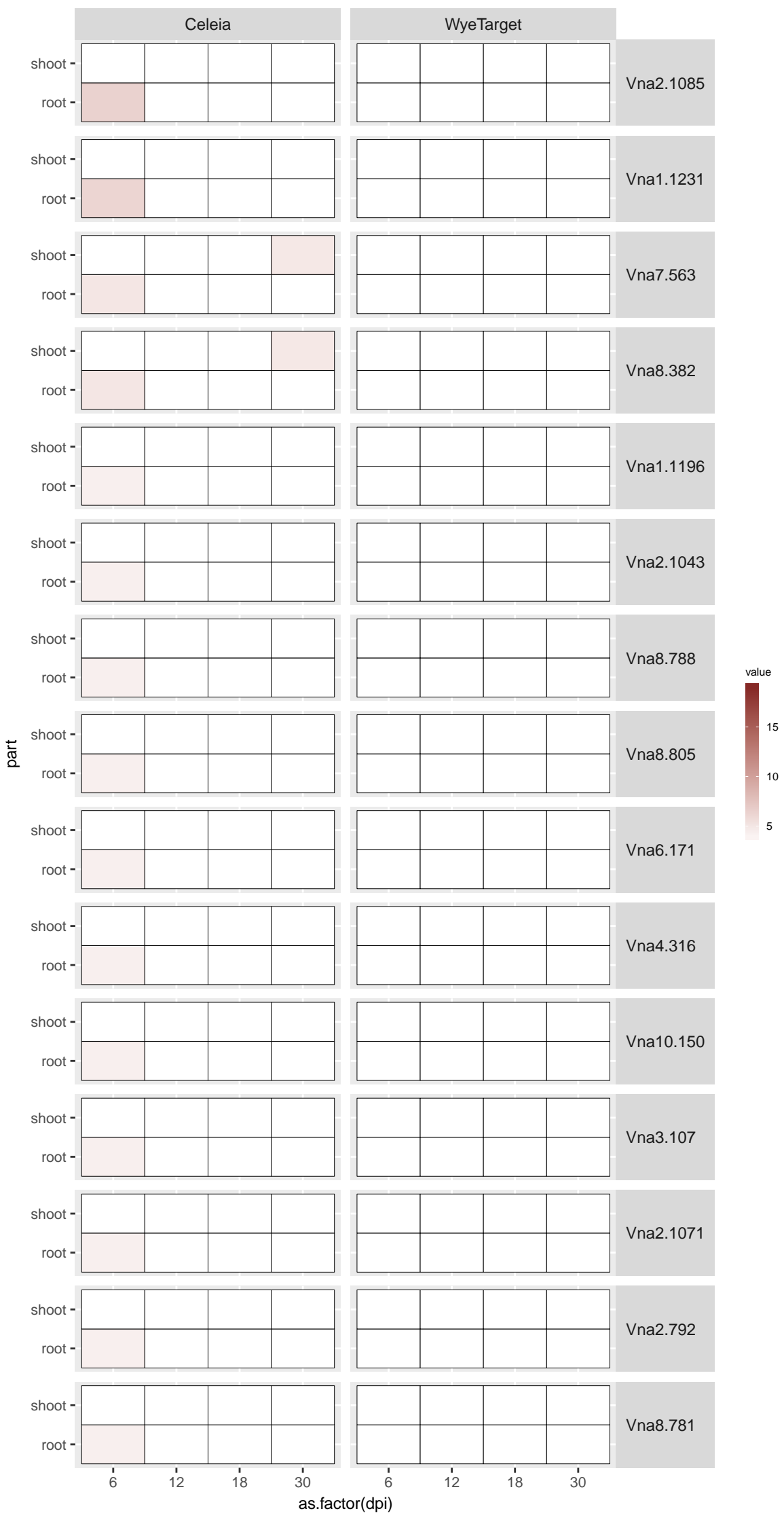

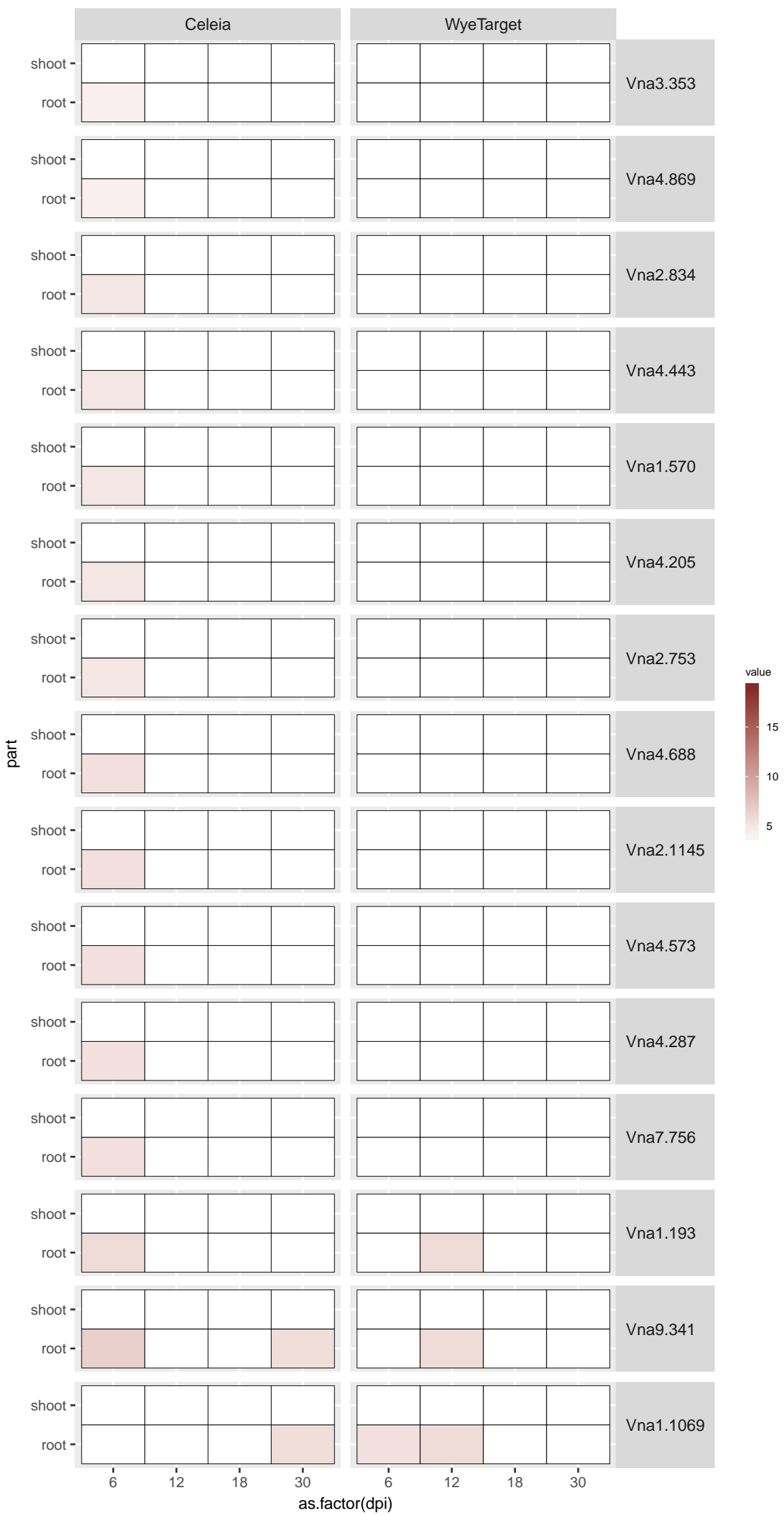

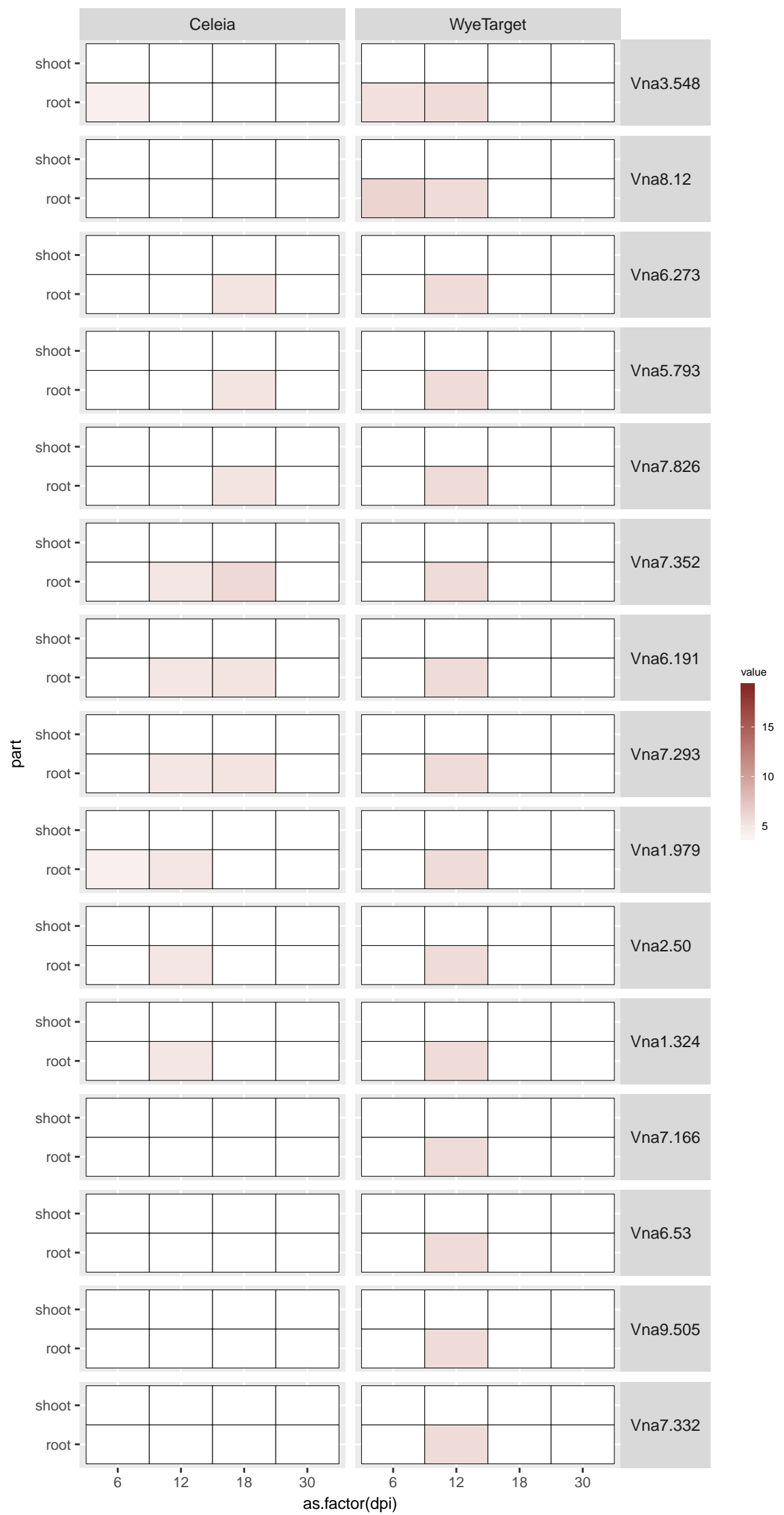

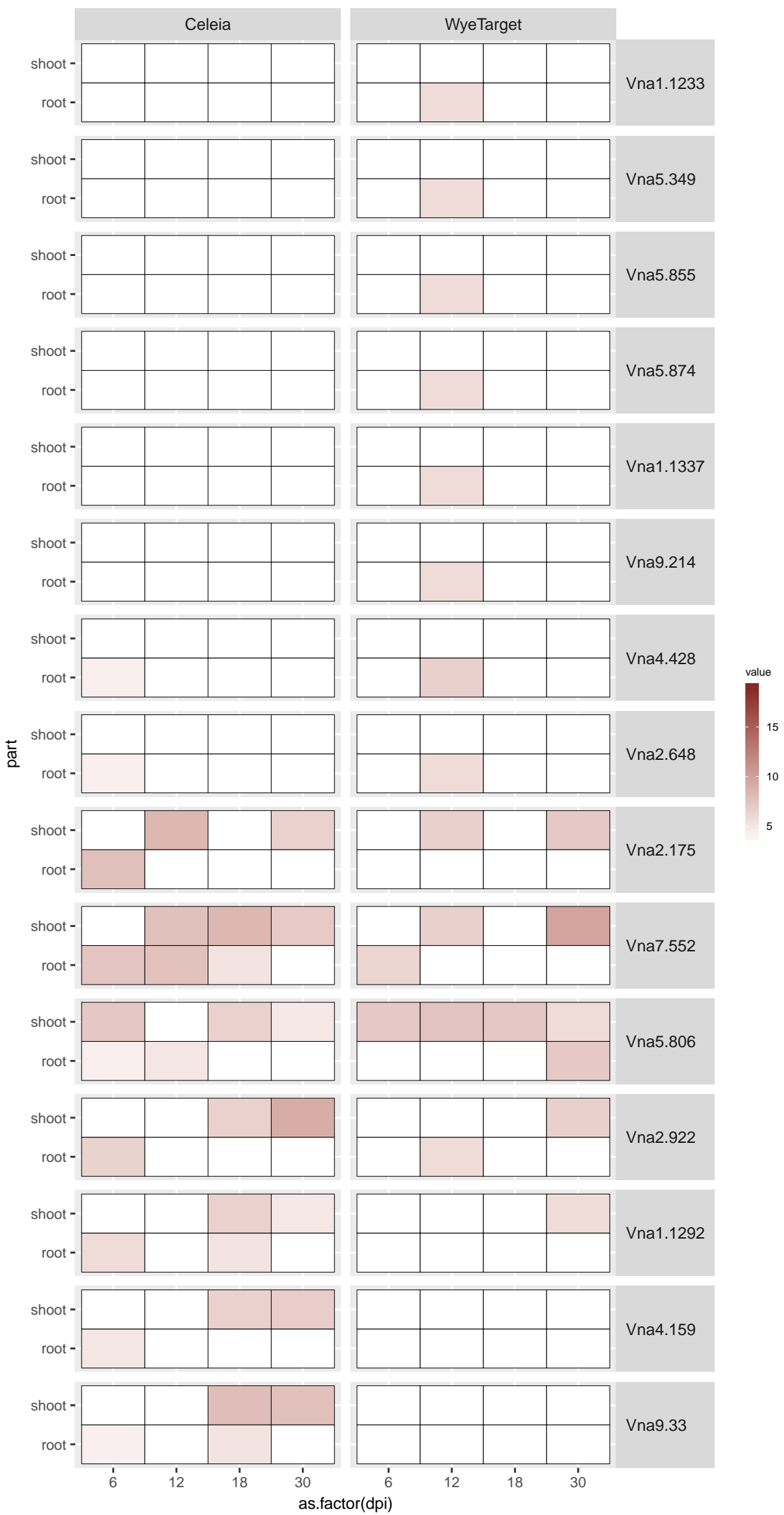

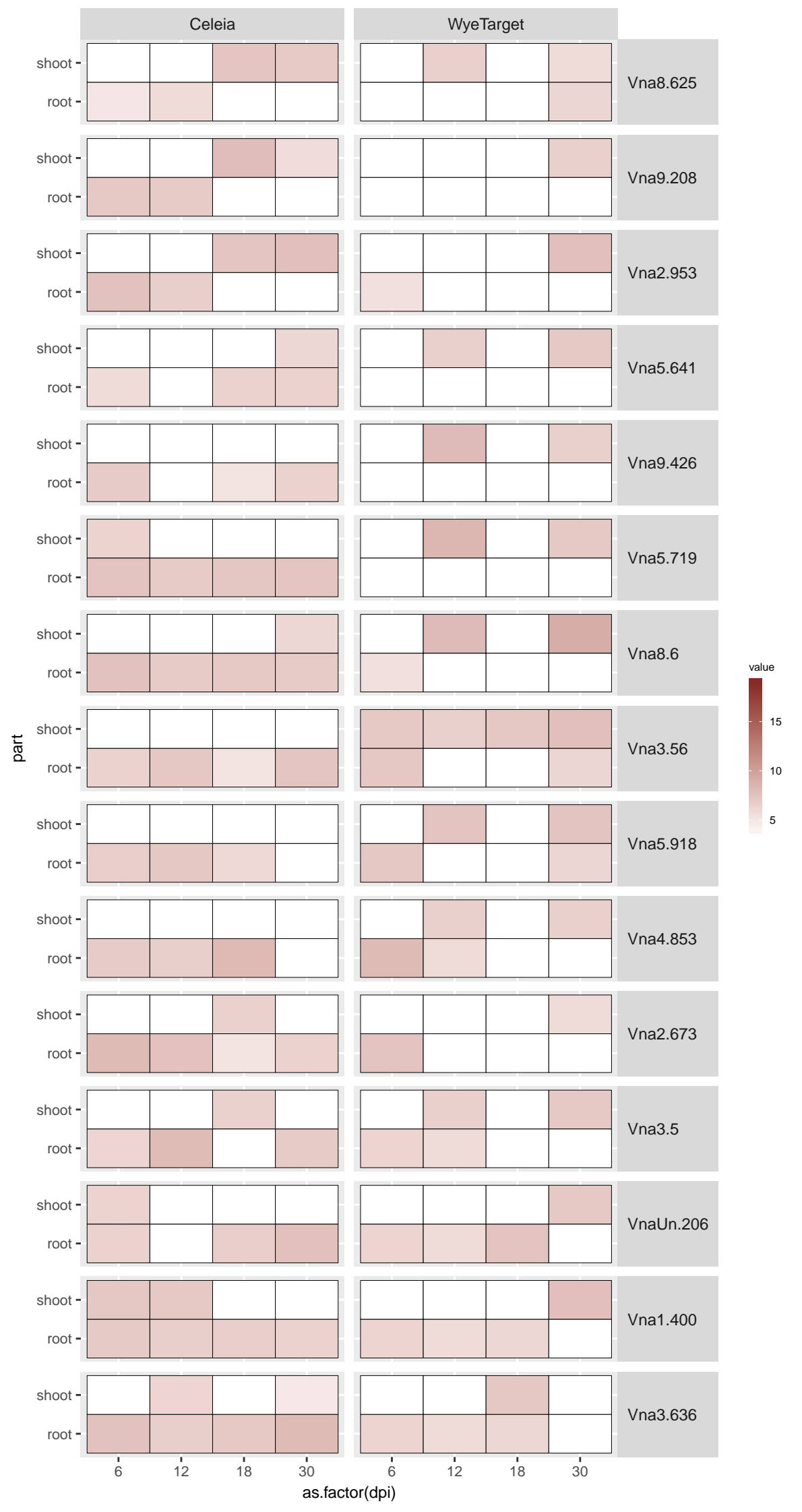

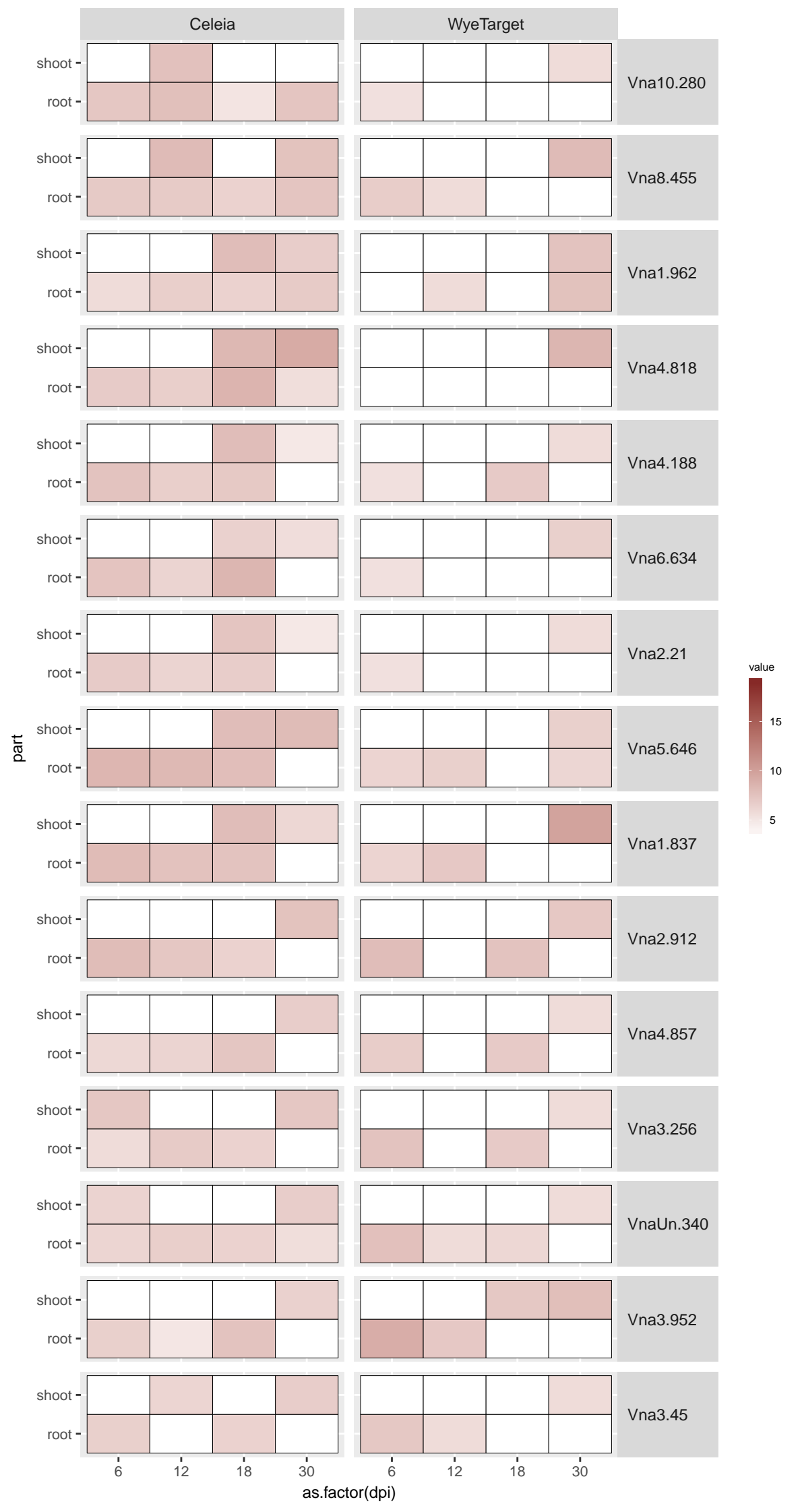

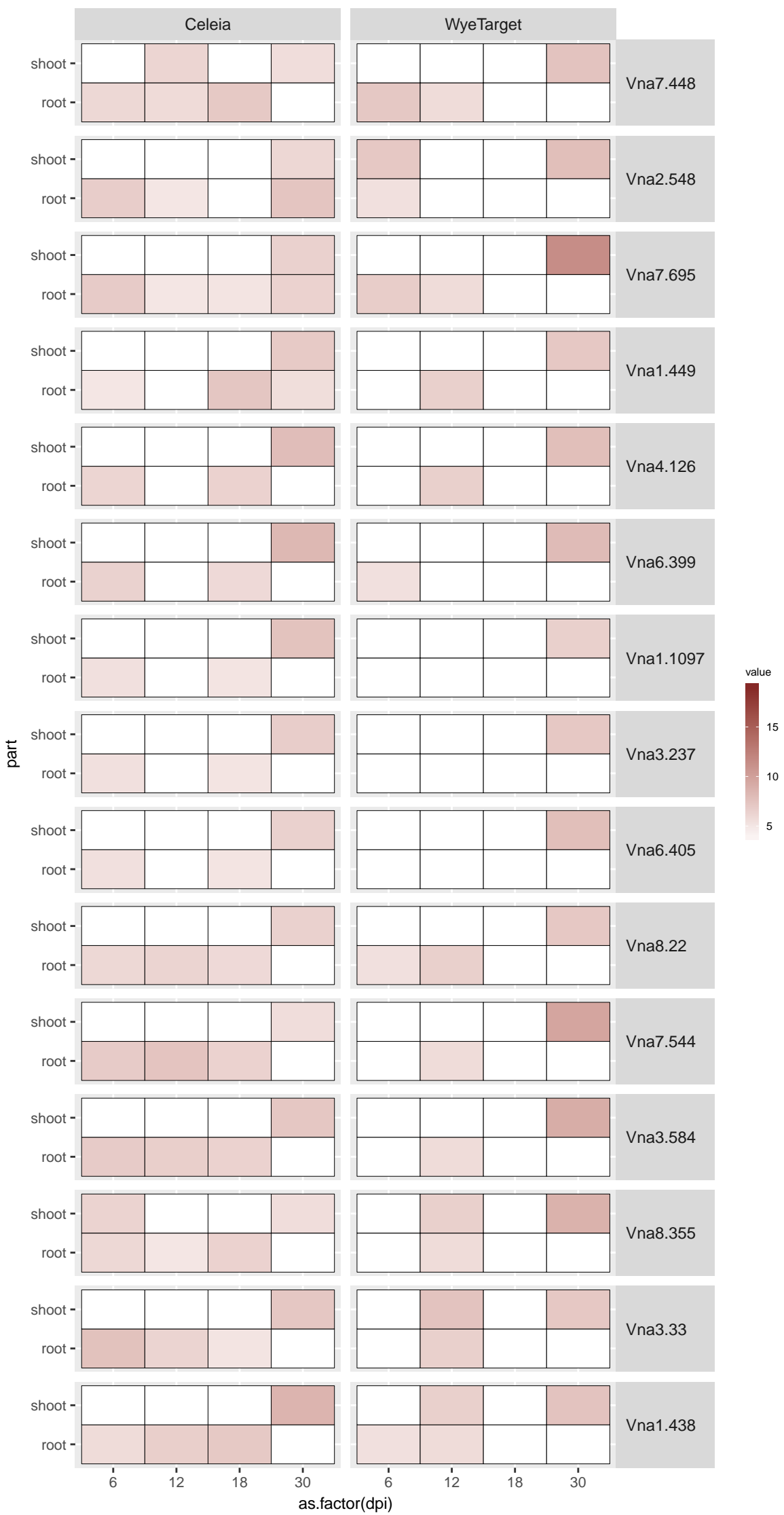

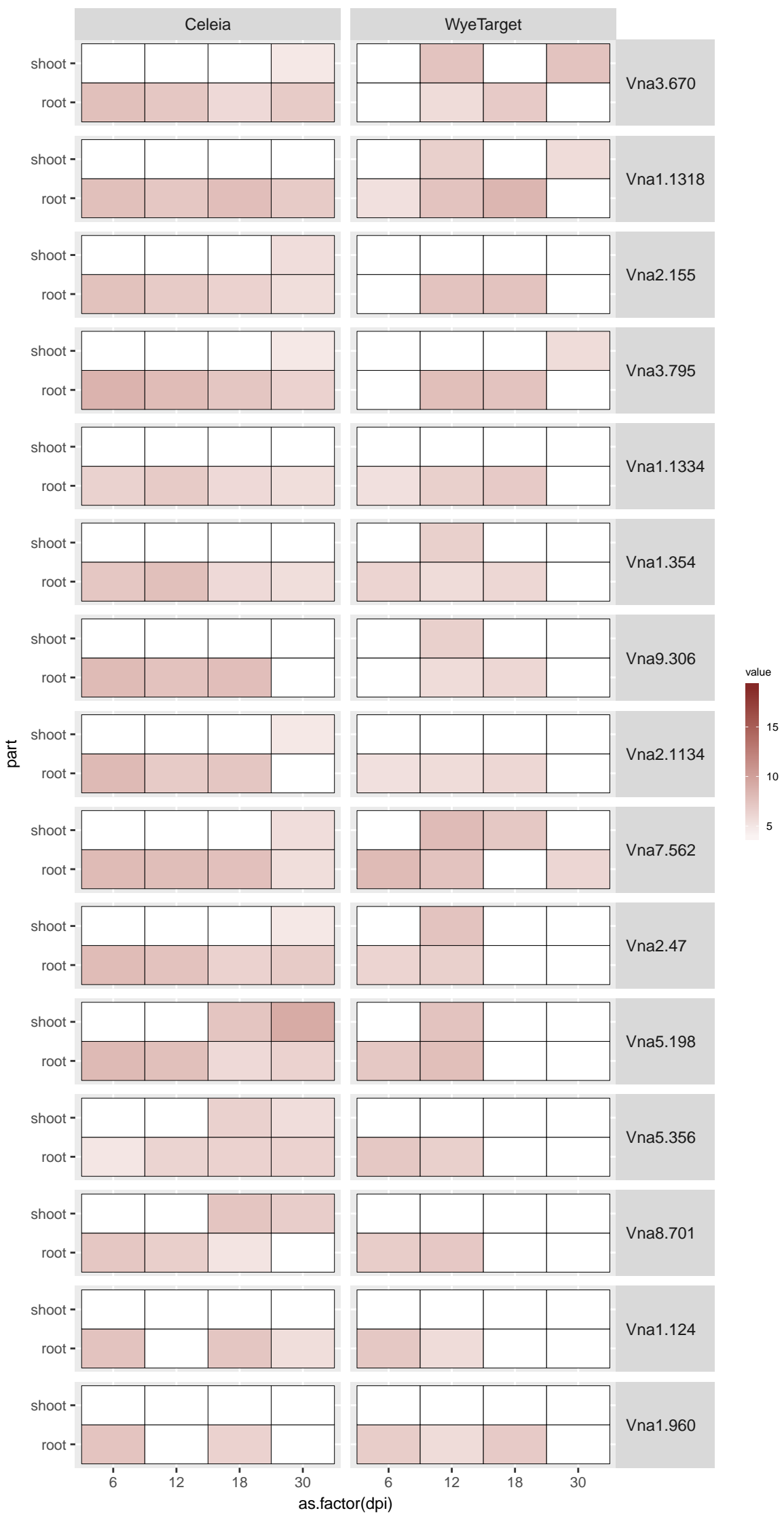

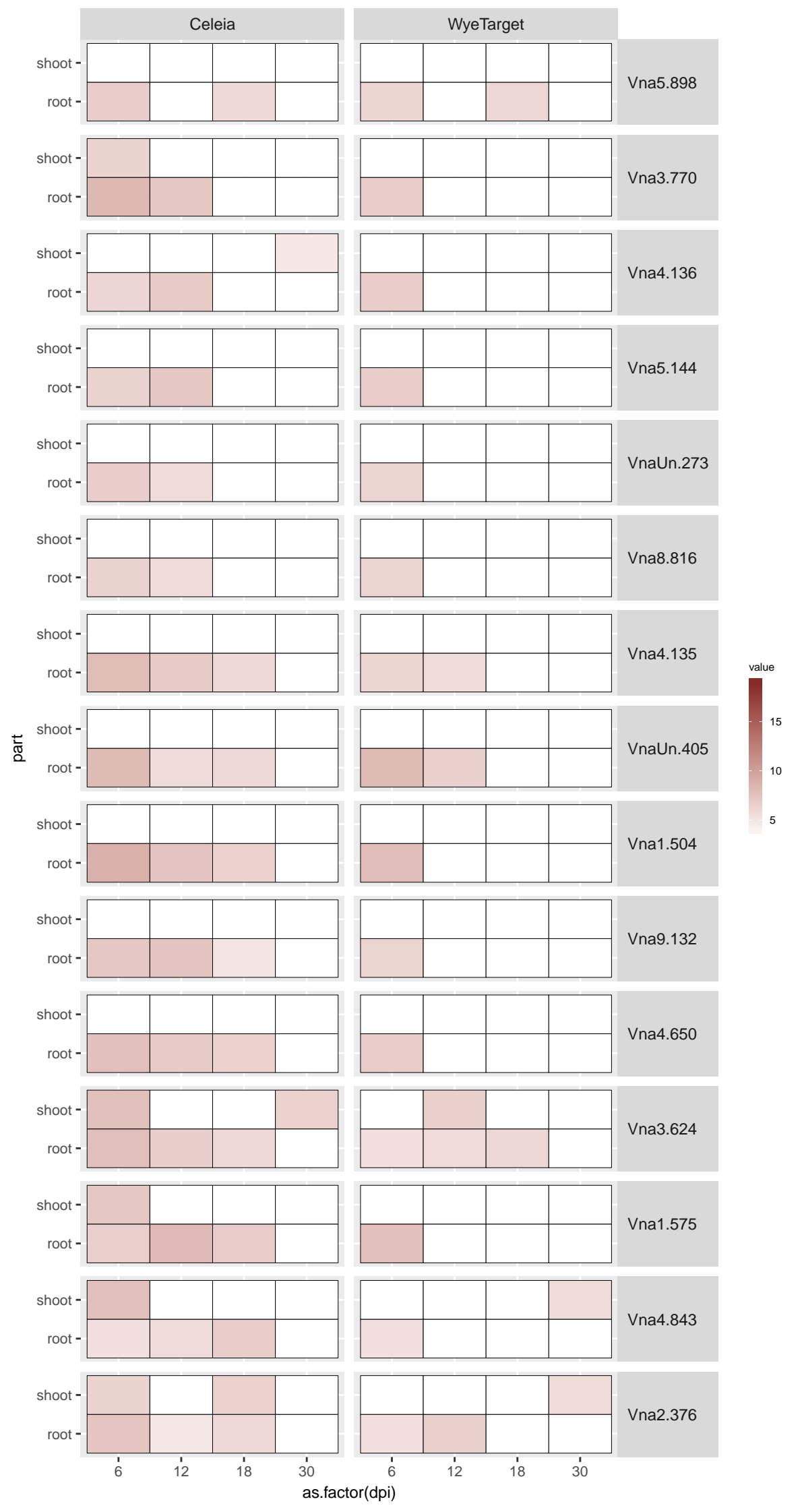

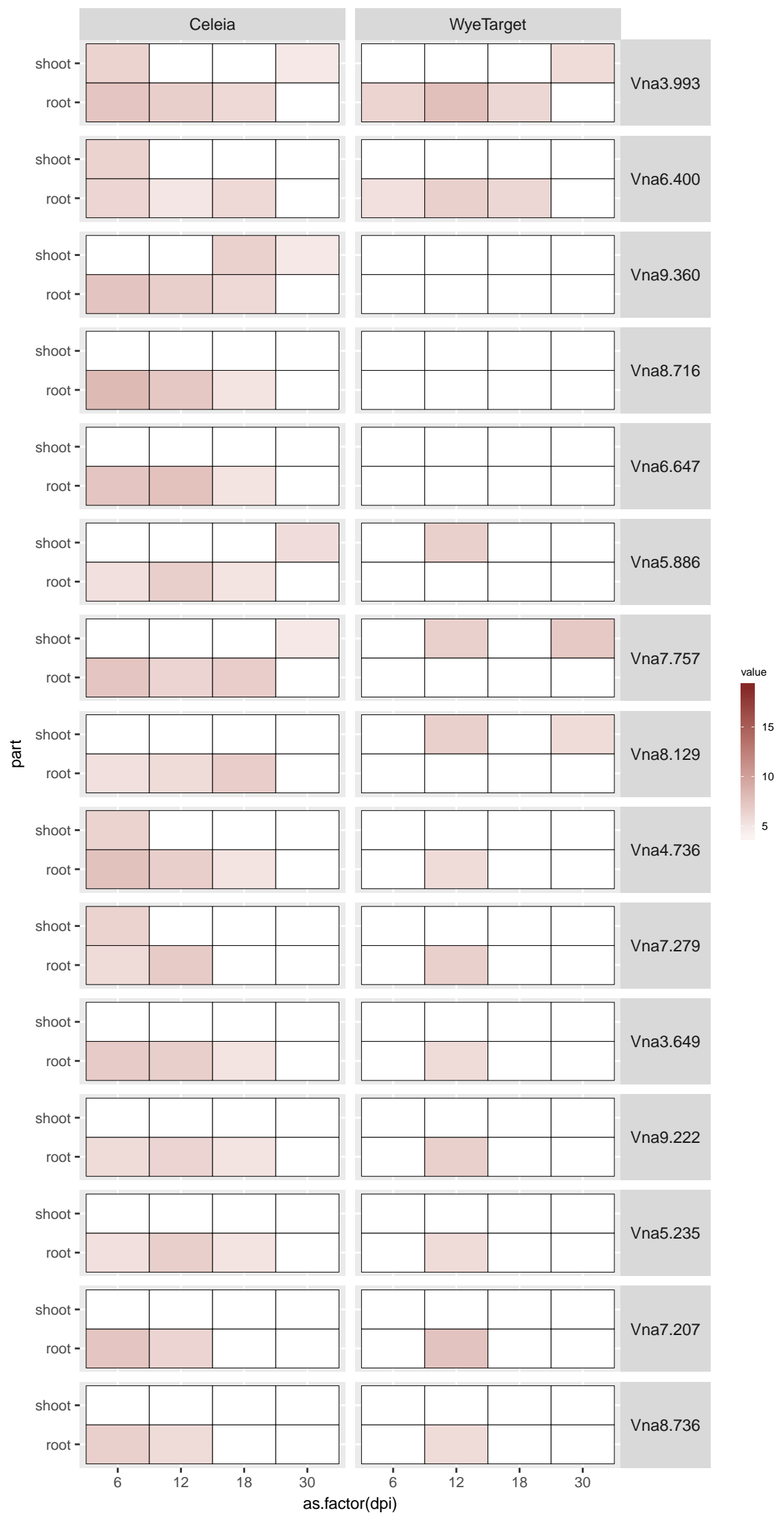

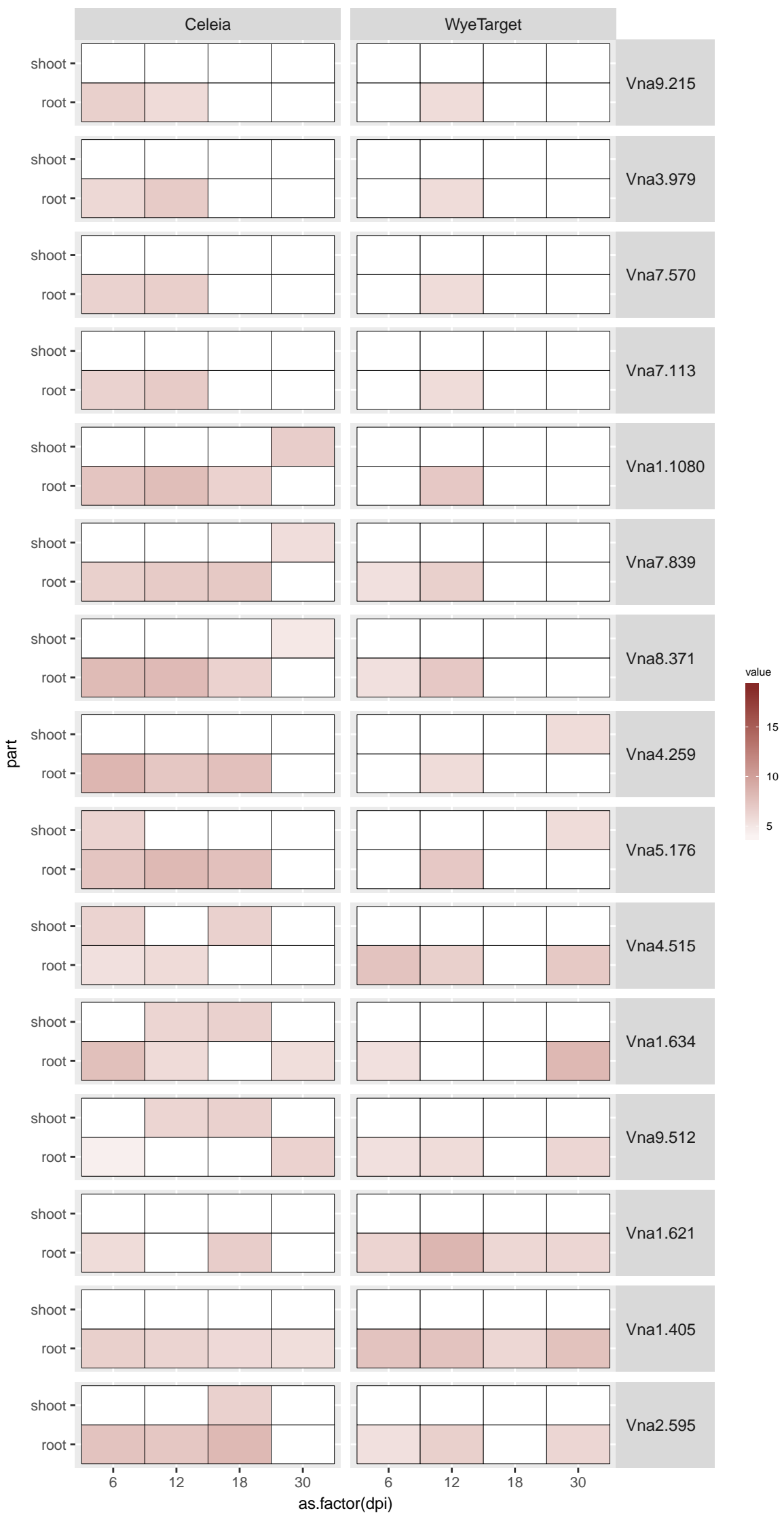

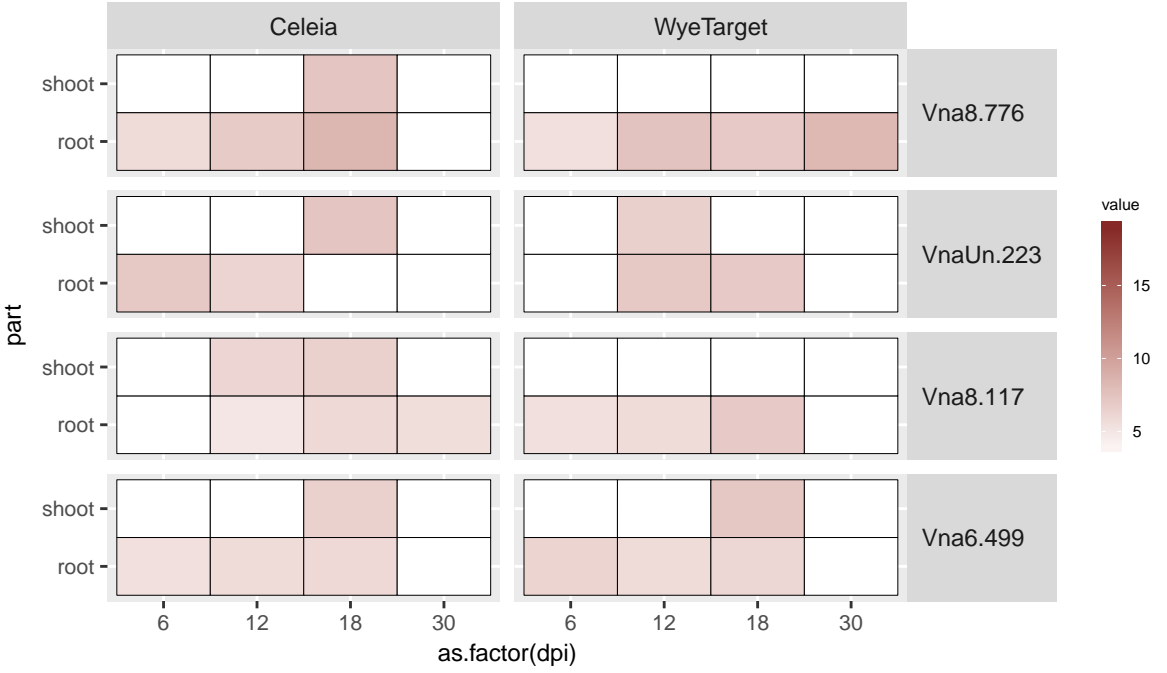

Supplement: S2 Fig — Fungal transcripts were first identified by mapping of reads with at least 90% sequence identity and 90% sequence coverage to the V. nonalfalfae reference genome [22] using CLC Workbench. Normalization by trimmed mean of M values (TMM) [129] was performed to eliminate composition biases between libraries. Read counts were converted into log2-counts-per-million (logCPM) values and a cutoff of CPM >1 was chosen. Color scale bar represents the logCPM values, with darker red color meaning higher expression values. (PDF) [file pone.0198971.s010.pdf]
